# Handgrip strength in children, adolescents, and young adults with suspected myalgic encephalomyelitis/chronic fatigue syndrome

**DOI:** 10.1186/s12967-026-08654-5

**Published:** 2026-07-15

**Authors:** Lorenz Mihatsch, Lisa Schartner, Julia Lange de Luna, Chiara Höhler, Lara Bucka, Lea Lovrenovic, Sophie Eidenschink, Birgit Schmuck, Viktoria Bienemann, Catharina Christa, Kirstin Mittelstraß, Anna Hausruckinger, Cordula Warlitz, Kaja Michel, Katrin Gerrer, Helma Freitag, Carmen Scheibenbogen, Rafael Pricoco, Uta Behrends

**Affiliations:** 1 Technical University of Munich; TUM School of Medicine and Health, Children’s Hospital, Munich Chronic Fatigue Center for Young People (MCFC), Munich, Germany; 2 Charité – Universitätsmedizin Berlin, corporate member of Freie Universität Berlin and Humboldt-Universität zu Berlin, Institute of Medical Immunology, Augustenburger Platz 1, Berlin, 13353 Germany; 3 German Center for Infection Research, Partnersite Munich, Munich, Germany

**Keywords:** Handgrip strength, ME/CFS, Pediatrics, Post-COVID, Post-Vaccination, Post-Exertional malaise, PEM

## Abstract

**Background:**

Myalgic encephalomyelitis/chronic fatigue syndrome (ME/CFS) in children and young people (CYP) lacks validated diagnostic biomarkers. Post-exertional malaise (PEM) is central to case definitions and is usually assessed by patient report. We evaluated the feasibility and clinical value of handgrip strength (HGS) testing in PEM-reporting CYP referred for suspected ME/CFS.

**Methods:**

In this prospective observational study at the Munich Chronic Fatigue Center for Young People (November 2022–November 2024), 147 patients (10–25 years) referred for the assessment of ME/CFS with positive DSQ-PEM screening and 83 healthy controls (HC) completed two HGS sessions (10 maximal grips/session; 3-s contraction/5-s rest; 60-minute inter-session break) using a digital dynamometer. We derived maximal force (Fmax), mean force (Fmean), fatigue ratio (FR = Fmax/Fmean), and recovery ratio (RR = Fmean session 2 / session 1). Analyses used repeated-measures ANCOVA, linear regression, partial Spearman correlations (adjusted for sex, age, and BMI), and proportional odds models for group membership (HC, noME/CFS, ME/CFS), reporting accuracy, and the C-statistic. Sensitivity analyses compared noME/CFS with confirmed CCC-ME/CFS.

**Results:**

After clinical work-up, 84/147 (57%) patients were classified as ME/CFS (confirmed or probable) and 63/147 (43%) as noME/CFS. HGS test completion rate was high (session 1: 146/147, 99.3%; session 2: 142/147, 96.6%). Compared with HC, patients had substantially lower HGS (mean difference −9.93 kg, 95% CI: −12.00 to −7.85), and HGS indices correlated modestly with physical functioning (SF-12 PCS), but not with PEM duration. Both noME/CFS and ME/CFS groups differed from HC in absolute strength indices (Fmean, Fmax) and FR. RR differed between ME/CFS and HC, whereas no HGS index significantly separated noME/CFS from ME/CFS. In proportional odds models, each HGS index improved fit (all *p* < 0.001), but discrimination across HC, noME/CFS, and ME/CFS patients was moderate (accuracy 49.2–57.3% vs no-information rate 36.5%, with best performance for Fmean in session 2). In the CCC-restricted sensitivity analysis, discrimination between confirmed CCC-ME/CFS and noME/CFS was moderate (accuracy 62.8–70.7%; C-statistic 0.63–0.73), with best performance for absolute strength indices and RR.

**Conclusions:**

Standardized two-session repeated HGS testing is feasible in CYP with chronic fatigue and self-reported PEM and provides an objective marker of functional impairment that aligns with physical health status but not with PEM duration. However, HGS alone shows limited ability to discriminate ME/CFS from other fatiguing noME/CFS conditions. HGS may be useful for quantitative phenotyping, patient stratification, and longitudinal outcome assessment rather than as a standalone diagnostic biomarker.

**Trial registration:**

Not applicable.

**Supplementary Information:**

The online version contains supplementary material available at 10.1186/s12967-026-08654-5.

## Background

Myalgic encephalomyelitis/chronic fatigue syndrome (ME/CFS) is a prevalent and debilitating multi-system disease that profoundly impacts daily life and participation of children, adolescents, and young adults (CYP) [1, 2]. Characterized by severe, persistent fatigue that is not alleviated by rest, and a range of other symptoms, ME/CFS is classified as a neurological disorder by the World Health Organization (WHO) [3] and is frequently triggered by acute viral infectious diseases.

In addition to long-lasting physical and mental fatigue, post-exertional malaise (PEM), unrefreshing sleep, cognitive impairment, and orthostatic intolerance are considered the hallmark features of ME/CFS and can be associated with pain, other autonomic, neuroendocrine, and flu-like symptoms. PEM refers to the exacerbation of symptoms and impaired recovery after daily activities that were well tolerated prior to the disease onset [1, 4].

In the absence of specific diagnostic biomarkers, diagnosing ME/CFS remains challenging and requires a comprehensive, symptom-oriented, differential diagnostic workup, as well as the assessment of established clinical criteria [1, 4, 5]. Furthermore, no objective marker of disease severity has been established. Although PEM is required by all major diagnostic criteria, its assessment is based on patient-reported responses in structured questionnaires and medical interviews, as objective functional testing may worsen the patient’s condition and is therefore not sufficiently feasible in many patients [6, 7]. Additionally, PEM-like symptoms were also reported in other fatiguing chronic conditions such as multiple sclerosis [8], fibromyalgia [9], cancer-related fatigue [10], major depression [8, 11, 12], and other post-acute infection or vaccination syndromes (PAIVS). Thus, although self-reported PEM, as assessed by the DePaul Symptom Questionnaire for PEM, has high discriminatory value for distinguishing ME/CFS from healthy controls [13], a specific diagnostic biomarker is not yet available.

Handgrip strength (HGS) is an objective and reproducible measure of fatigability, muscle function, and overall health status [14]. In adult patients with ME/CFS and post-COVID condition (PCC), it has, thus, been proposed as a potential diagnostic tool, a candidate measure of disease severity, and a monitoring and outcome indicator in several studies [15–20].

### Rationale for HGS testing in children and young people with suspected ME/CFS

In CYP, HGS is a promising diagnostic biomarker for ME/CFS and a marker of disease severity. Its objectivity, low exercise burden, and the portability of measurement equipment make it particularly attractive, minimizing the risk of provoking PEM. Reduced HGS in CYP correlates with adverse metabolic and general health outcomes and has proven utility as a disease activity and disability indicator in other pediatric health conditions [21–24]. Importantly, beyond absolute muscular strength, intra-session decline across repeated grips may indicate fatigability, and missing inter-session recovery in grip strength may indicate reduced recoverability. Both fatigability and reduced recoverability are central features of ME/CFS, and associated handgrip strength patterns have been observed in adult patients with ME/CFS and PCC [15–20]. Taken together, a standardized HGS protocol may offer a pragmatic and scalable tool to support differential diagnosis in CYP with suspected ME/CFS who suffer from PEM.

### Objectives

We aimed to i) assess the feasibility of standardized HGS testing in CYP with self-reported PEM referred for the evaluation of suspected ME/CFS, ii) quantify associations between HGS and ME/CFS severity, and iii) evaluate the potential of HGS as a diagnostic tool to distinguish ME/CFS from other fatiguing conditions (noME/CFS) and healthy controls (HC).

## Material & methods

### Study design and population

This prospective observational study included 147 patients with suspected PAIVS and self-reported PEM referred for the evaluation of ME/CFS, as well as 83 healthy controls (HC). The study was conducted between November 2022 and November 2024 at the Munich Chronic Fatigue Centre for Young People (MCFC) of the Technical University of Munich (TUM).

### Ethics

The study was approved by the Institutional Review Board of the TUM University Hospital (2022-585_1) and adheres to the Declaration of Helsinki and its subsequent amendments. Written informed consent was obtained from all participants prior to their inclusion, and in the case of minors, from their legal guardians.

### Inclusion and exclusion criteria

Inclusion criteria for patients were i) age 10 to 25 years, ii) suspected ME/CFS, iii) a positive PEM-screening using the DSQ-PEM questionnaire [25], and iv) written informed consent. Exclusion criteria were i) any known pre-existing condition likely to interfere with valid study participation (e.g., injury or malformation of extremities) or likely to prevent reliable responses to questionnaires (e.g., intellectual disability, intensive care treatment, language problems), and ii) pregnancy or breastfeeding.

Inclusion criteria for HC were i) age 10 to 25 years, ii) being clinically healthy without relevant known pre-existing conditions (asthma, allergies, or atopic dermatitis were eligible if these conditions did not require regular medication), and iii) written informed consent. Exclusion criteria for HC were i) known acute or chronic diseases (except those named above), ii) pregnancy or breastfeeding, and iii) any condition likely to interfere with valid study participation.

### Standardized handgrip strength testing protocol

HGS was measured with a digital hand dynamometer (EH101, Deyard, Shenzhen, China) according to the standardized HGS protocol by Jäkel et al. [16]. Patients were instructed to sit upright and position the forearm of their dominant hand on a table in full supination. Handle position was adapted to hand size. The handle was pulled with maximum force for three seconds, followed by a five-second relaxation phase. Within each of two consecutive sessions, this procedure was repeated ten times. The second session was conducted after a 60-minute break following the end of the first session without any strenuous physical activity. Participants were verbally encouraged throughout the measurements to use their maximum strength and complete all required repetitions.

Per session, three HGS indices were calculated: the maximum force (Fmax [kg]), the mean force (Fmean [kg]), and the fatigue ratio (FR), a parameter defined as the quotient of Fmax and Fmean.

Higher FR values indicate a stronger decrease in force within a single session. Additionally, the recovery ratio (RR), defined as the ratio of the Fmean achieved in the second session to that achieved in the first session, was used as an indicator for impaired recovery [16].

### Collection of clinical data based on patient-reported outcomes

Demographics and clinical characteristics were assessed upon study inclusion in a semi-structured multiprofessional medical interview. Functional impairment (Bell Score), health-related quality of life (HRQoL) (SF12 Physical Component Summary (SF12-PCS) and Mental Health Component Summary (SF12-MCS) scores), fatigue severity (FSS), and self-reported PEM (DSQ-PEM) were assessed using validated instruments [25–28].

### ME/CFS diagnosis

ME/CFS (ICD-10 GM G93.3) was diagnosed following a thorough differential diagnostic work-up, including the semi-structured medical and psychological interview, and according to clinical criteria. Fulfilment of at least one of four sets of clinical criteria with obligatory PEM, evaluated by the Munich Berlin Symptom Questionnaire (MBSQ, see below) [5], was required for a confirmed diagnosis. Patients were categorized as i) ME/CFS patients, when ME/CFS was confirmed or highly suspected (probable), or ii) noME/CFS patients, when ME/CFS was excluded. The MBSQ was developed to allow a structured diagnostic approach based on four published sets of diagnostic clinical ME/CFS criteria, including the Canadian Consensus criteria (CCC) [29] and the criteria of the former Institute of Medicine (IOM) [3] for all patients and, additionally, the Pediatric Case Definition by Jason et al. (PCD-J) [30] and the Clinical Diagnostic Worksheet by Rowe et al. (CDW-R) [1] for patients ≤ 17 years of age [31].

### Post-COVID and post-vaccination condition

For the diagnosis of a Post-COVID Condition (PCC), the WHO definition for adult patients [32] or, in the case of patients aged ≤ 17 years, the WHO definition for children was applied [33, 34]. An analogous definition was used to diagnose a post-COVID-19 vaccination condition (PVC) [33].

### Statistical analyses

Continuous variables are summarized as mean ± SD and/or (median; IQR); Min and Max, as appropriate. Categorical variables are presented as absolute (%) frequencies. Group comparisons were performed using Pearson’s Chi-squared tests, Fisher’s exact tests, Kruskal-Wallis rank sum tests, and Wilcoxon’s rank sum tests, as appropriate.

#### Marginal means analysis

HGS measurements, stratified by sex, were analysed using repeated-measures ANCOVA adjusted for age and BMI. For each sex, marginal means of force across ten repeated measurements were estimated separately for patients and HC, stratified by session.

In a separate analysis, each HGS index (Fmax, Fmean, and FR of both sessions as well as the RR) was examined individually using linear regression models. Models were adjusted for sex, age, and body mass index (BMI). Pairwise post-hoc comparisons among HC, ME/CFS, and noME/CFS patients were conducted using Tukey’s method corrected for multiple testing.

To reduce diagnostic heterogeneity within the ME/CFS group, all marginal means models were rerun comparing noME/CFS patients exclusively to confirmed ME/CFS according to the CCC (CCC-ME/CFS).

#### Partial correlations

Partial Spearman correlations were estimated using the *ppcor* R-package to quantify the associations between HGS parameters and Patient-Reported Outcome Measures (PROMs) [35]. For each pair of variables, raw (unadjusted) and partial (adjusted) correlations were estimated. Partial correlations were adjusted for sex, age, and BMI.

#### Proportional odds models

We used proportional odds regression (probit link) to model diagnostic group membership (HC, noME/CFS, ME/CFS) as an ordinal outcome. Each HGS index was entered into a separate model, adjusted for sex, age, and BMI. Model contributions were tested using likelihood ratio tests (LRTs) comparing full models with reduced models that excluded the HGS index. Performance was quantified by overall accuracy and C-statistic, as well as sensitivity, specificity, positive and negative predictive value (PPV, NPV) were additionally computed for each class. Performance measures were stratified by age group (10–12, 13–17, 18–25 years). Marginal predicted probabilities were estimated with *emmeans* R-package for visualization. In sensitivity analyses, models were refitted comparing noME/CFS with confirmed CCC-ME/CFS, and LRT, accuracy, and C-statistic were reassessed, as were sensitivity, specificity, PPV, and NPV.

All statistical analyses were conducted in R, version 4.2.1 (“Angel Food Cake”; The R Foundation for Statistical Computing, Vienna, Austria). The level of significance was set at α = 0.05. *p*-values were indicated as follows: *p* < 0.1 (.), *p* < 0.05 (*), *p* < 0.01 (**), *p* < 0.001 (***).

## Results

### Demographics and clinical characteristics of patients and healthy controls

All patients (*N* = 147) had a positive DSQ-PEM screening and were referred for evaluation of suspected ME/CFS. Detailed clinical characteristics of patients and HC (*N* = 83) are shown in Table 1. The HC group included significantly more young adults and fewer adolescents compared to the patient group.

**Table 1:** Demographics and clinical characteristics of patients and healthy controls

Characteristic	Healthy Controls *N* = 83^1^	Patients *N* = 147^1^	**P-Val.**^2^
Sex			0.490
Male	32 / 83 (39%) [0]	50 / 147 (34%) [0]	
Female	51 / 83 (61%) [0]	97 / 147 (66%) [0]	
Age Group			0.001**
Children (10-12y)	10 / 83 (12%) [0]	18 / 147 (12%) [0]	
Adolescents (13-17y)	31 / 83 (37%) [0]	90 / 147 (61%) [0]	
Young Adults (18-25y)	42 / 83 (51%) [0]	39 / 147 (27%) [0]	
BMI [kg/m^2^]	21.1 ± 3.1 (21.2; 18.9–23.0); Min: 11.5, Max: 30.1 [0]	21.0 ± 4.1 (20.3; 18.4 - 22.8); Min: 11.6, Max: 34.6 [0]	0.377
**Patient-Reported Outcome Measures (PROMs)**			
Bell Score	98 ± 4 (100; 100–100); Min: 90, Max: 100 [0]	40 ± 15 (40; 30 - 50); Min: 10, Max: 90 [0]	<0.001***
Bell Score Lowest^3^	-	19 ± 12 (20; 10 - 30); Min: 0, Max: 60 [12]	-
Bell Score Highest^3^	-	54 ± 19 (50; 40 - 70); Min: 20, Max: 100 [12]	-
SF12 - PCS	56 ± 3 (56; 55 - 57); Min: 45, Max: 63 [11]	27 ± 10 (26; 18 - 33); Min: 11, Max: 57 [18]	<0.001***
SF12 - MCS	52 ± 6 (54; 49 - 57); Min: 35, Max: 61 [11]	46 ± 10 (49; 40 - 54); Min: 21, Max: 64 [18]	<0.001***
Fatigue Severity Scale	1.91 ± 0.87 (1.67; 1.22 - 2.44); Min: 1.00, Max: 4.56 [45]	6.35 ± 0.70 (6.56; 6.11 - 6.89); Min: 2.78, Max: 7.00 [0]	<0.001***
DSQ-PEM Screening			-
negative	81 / 83 (98%) [0]	0 / 147 (0%) [0]	
positive	2 / 83 (2.4%) [0]	147 / 147 (100%) [0]	
DSQ-PEM Duration			-
<1 h	54 / 76 (71%) [7]	2 / 147 (1%) [0]	
2 - 3 h	20 / 76 (26%) [7]	16 / 147 (11%) [0]	
4 - 10 h	2 / 76 (2.6%) [7]	20 / 147 (14%) [0]	
11 - 13 h	0 / 76 (0%) [7]	15 / 147 (10%) [0]	
14 - 23 h	0 / 76 (0%) [7]	21 / 147 (14%) [0]	
>24 h	0 / 76 (0%) [7]	72 / 147 (50%) [0]	
**ME/CFS Diagnostic Criteria (MBSQ)**			
CCC fulfilled	0 / 76 (0%) [7]	58 / 147 (39%) [0]	-
IOM Criteria fulfilled	0 / 76 (0%) [7]	79 / 147 (54%) [0]	-
CDW-R Criteria fulfilled^4^	0 / 41 (0%) [0]	43 / 107 (40%) [1]	-
PCD-J Criteria fulfilled^4^	0 / 41 (0%) [0]	39 / 107 (36%) [1]	-
**Diagnoses**			
ME/CFS (ICD-10 G93.3)			-
excluded	83 / 83 (100%) [0]	63 / 147 (43%) [0]	
probable^5^	0 / 83 (0%) [0]	30 / 147 (20%) [0]	
confirmed	0 / 83 (0%) [0]	54 / 147 (37%) [0]	
Post-COVID (U09.9!)^6^	0 / 83 (0%) [0]	77 / 147 (52%) [0]	-
Post-VAC (U12.9!)^6^	0 / 83 (0%) [0]	8 / 147 (5%) [0]	-

^1^n/N (%) [# Missing] or Mean ± SD (Median; Q1 - Q3); Min, Max [# Missing]

^2^Pearson’s Chi-squared test; Wilcoxon rank sum test

^3^Only applied on patients, not on healthy controls

^4^CDW-R and PCD-J were only assessed in children and adolescents below 18 years of age. Number of missing values refer to the number of patients below 18 years of age

^5^Most likely diagnosis after extensive diagnostic work-up

^6^Patients with post-COVID-19 condition (U09.9!) and/or post-COVID-19 vaccination condition (U12.9!)

*p*-values are indicated by *p* < 0.1(.), *p* < 0.05(*), *p* < 0.01(**), *p* < 0.001(***)

Abbreviations: BMI - Body Mass Index; ME/CFS - myalgic encephalomyelitis / chronic fatigue syndrome; SF12-PCS - Short Form 12 Health Survey (SF12) Physical Component summary scale; SF12-MCS - SF12 Mental Health Component summary scale; PEM - Post-Exertional Malaise; CCC - Canadian Consensus Criteria; IOM - Institute of Medicine; CDW-R - Clinical Diagnostic Worksheet by Rowe PC et al. (2017); PCD-J - Pediatric Case Definition by Jason LA et al. (2011)

After comprehensive clinical evaluation, 84/147 (57%) patients were diagnosed with ME/CFS (ICD-10 GM G93.3 confirmed or probable). In 63/147 (43%) patients, the ME/CFS diagnosis was excluded. A detailed comparison of demographic and clinical characteristics of ME/CFS, noME/CFS patients, and HC can be found in Suppl. Table S1. Most frequent diagnoses recorded in the noME/CFS disease-control group are summarized in Suppl. Table S2.

### Feasibility of standardized handgrip strength in patients with post-exertional malaise

**Fig. 1:**
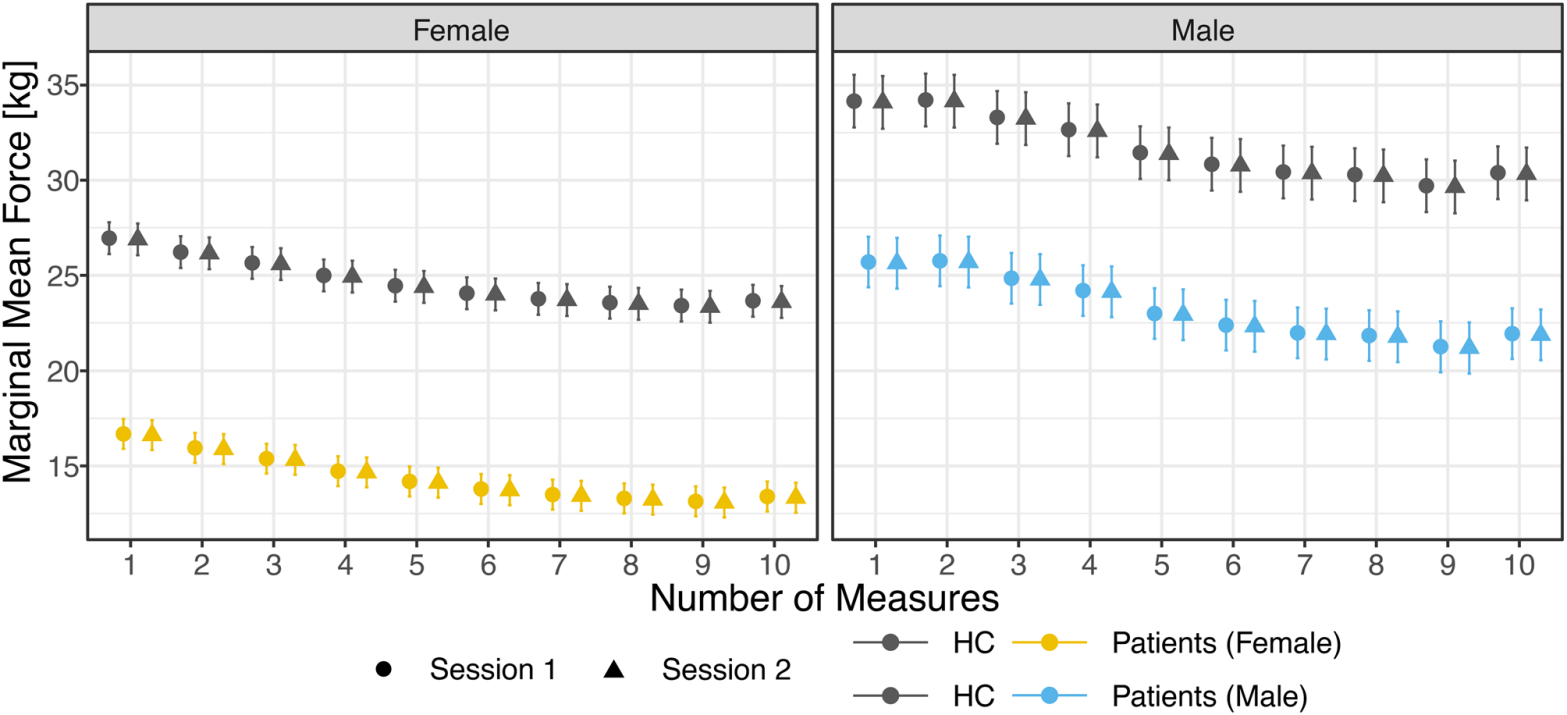
Marginal mean of hand grip strength (HGS) across ten repeated measures, stratified by sex, session, and group (healthy controls versus patients). Estimates are based on a linear model with measure, session, group, age, and body mass index (BMI) as predictors and strength (F) as the dependent variable. Error bars represent 95% confidence intervals

All 83 HC completed both sessions. Among patients, 146/147 (99.3%) completed the first session, and 142/147 (96.6%) the second session. Figure 1 shows the marginal mean HGS of HC and patients, stratified by sex, and adjusted for age and BMI. A repeated measures ANCOVA estimated a marginal mean difference in HGS between patients and HC of −9.93 kg (95% CI: −12.00 to −7.85), between males and females of 8.99 kg (95% CI: 6.99 to 10.99), and between the two sessions of −0.02 kg (95% CI: −0.36 to 0.33). From measurement to measurement, the marginal mean HGS declined by 0.58 kg (95% CI: −0.61 to −0.54) in HC, by 0.39 kg (95% CI: −0.43 to −0.34) in ME/CFS patients, and by 0.39 kg (95% CI: −0.44 to −0.35) in noME/CFS patients.

Table 2 shows the HGS indices stratified by sex and age group for HC and patients. In HC, Fmax and Fmean of both sessions significantly increased with age for both sexes. The FR of session 1, but not the FR of session 2, significantly decreased with age in female HC (*p* = 0.005**). For male HC, FR did not significantly change by age. The RR did not change by age group in either sex.

**Table 2:** Handgrip strength in healthy controls and patients stratified by sex and age

Healthy Controls								
Parameter	Male				Female			
	Children (10-12y) *N* = 5^1^	Adolescents (13-17y) *N* = 14^1^	Young Adults (18-25y) *N* = 13^1^	**p-Value**^**2**^	Children (10-12y) *N* = 5^1^	Adolescents (13-17y) *N* = 17^1^	Young Adults (18-25y) *N* = 29^1^	**p-Value**^**2**^
Fmax Session 1 in [kg]	28 ± 12 (23; 22 - 29); Min: 17, Max: 47 [0]	37 ± 10 (34; 32 - 39); Min: 25, Max: 62 [0]	46 ± 8 (46; 44 - 51); Min: 28, Max: 61 [0]	0.005**	23.9 ± 7.3 (21.2; 18.7 - 31.1); Min: 16.3, Max: 32.1 [0]	25.0 ± 5.2 (23.1; 21.8 - 30.0); Min: 17.7, Max: 33.8 [0]	29.6 ± 6.0 (29.5; 25.3 - 34.2); Min: 19.1, Max: 42.7 [0]	0.027*
Fmean Session 1 in [kg]	23 ± 10 (19; 18 - 22); Min: 15, Max: 41 [0]	32 ± 8 (30; 29 - 35); Min: 22, Max: 52 [0]	41 ± 8 (43; 38 - 46); Min: 23, Max: 53 [0]	0.002**	20.6 ± 5.8 (19.4; 17.3 - 26.3); Min: 13.4, Max: 26.8 [0]	21.5 ± 5.2 (21.3; 18.1 - 24.4); Min: 13.8, Max: 31.7 [0]	27.0 ± 5.3 (27.7; 23.3 - 31.2); Min: 17.5, Max: 37.9 [0]	0.003**
FR Session 1	1.19 ± 0.07 (1.16; 1.15 - 1.25); Min: 1.11, Max: 1.28 [0]	1.14 ± 0.06 (1.14; 1.12 - 1.16); Min: 1.07, Max: 1.29 [0]	1.13 ± 0.05 (1.14; 1.10 - 1.15); Min: 1.05, Max: 1.22 [0]	0.324	1.15 ± 0.07 (1.16; 1.09 - 1.22); Min: 1.08, Max: 1.22 [0]	1.18 ± 0.09 (1.18; 1.10 - 1.25); Min: 1.05, Max: 1.39 [0]	1.09 ± 0.05 (1.09; 1.06 - 1.11); Min: 1.04, Max: 1.29 [0]	0.005**
Fmax Session 2 in [kg]	26 ± 10 (24; 21–25); Min: 19, Max: 43 [0]	39 ± 11 (35; 33–45); Min: 23, Max: 66 [0]	47 ± 8 (48; 40–51); Min: 30, Max: 63 [0]	0.003**	24 ± 6 (23; 21 - 28); Min: 16, Max: 33 [0]	26 ± 6 (25; 20 - 30); Min: 17, Max: 37 [0]	31 ± 6 (30; 27 - 36); Min: 18, Max: 44 [0]	0.007**
Fmean Session 2 in [kg]	22 ± 9 (19; 19 - 20); Min: 16, Max: 37 [0]	32 ± 9 (30; 26 - 37); Min: 20, Max: 54 [0]	41 ± 9 (43; 34 - 47); Min: 23, Max: 54 [0]	0.002**	20.9 ± 4.6 (19.7; 18.9 - 24.4); Min: 15.1, Max: 26.5 [0]	22.4 ± 5.3 (22.0; 18.5 - 25.4); Min: 15.5, Max: 34.9 [0]	27.3 ± 5.2 (27.0; 23.6 - 31.1); Min: 16.5, Max: 37.1 [0]	0.003**
FR Session 2	1.18 ± 0.06 (1.16; 1.15 - 1.25); Min: 1.10, Max: 1.25 [0]	1.20 ± 0.07 (1.20; 1.15 - 1.22); Min: 1.05, Max: 1.32 [0]	1.15 ± 0.08 (1.16; 1.10 - 1.17); Min: 1.04, Max: 1.31 [0]	0.264	1.14 ± 0.06 (1.14; 1.10 - 1.15); Min: 1.09, Max: 1.23 [0]	1.14 ± 0.06 (1.12; 1.10 - 1.19); Min: 1.05, Max: 1.25 [0]	1.14 ± 0.08 (1.12; 1.10 - 1.19); Min: 1.03, Max: 1.36 [0]	0.957
Recovery Ratio	0.97 ± 0.08 (0.98; 0.91 - 1.05); Min: 0.88, Max: 1.05 [0]	1.00 ± 0.08 (1.02; 0.92 - 1.05); Min: 0.87, Max: 1.15 [0]	1.00 ± 0.08 (1.00; 0.97 - 1.00); Min: 0.89, Max: 1.22 [0]	0.811	1.03 ± 0.08 (1.02; 0.99 - 1.09); Min: 0.93, Max: 1.13 [0]	1.05 ± 0.10 (1.01; 0.98 - 1.10); Min: 0.92, Max: 1.35 [0]	1.01 ± 0.08 (1.02; 0.96 - 1.04); Min: 0.86, Max: 1.22 [0]	0.642
**Patients**								
**Characteristic**	**Male**				**Female**			
	**Children (10-12y)** *N* = 11^1^	**Adolescents (13-17y)** *N* = 28^1^	**Young Adults (18-25y)** *N* = 11^1^	**p-Value**^**2**^	**Children (10-12y)** *N* = 7^1^	**Adolescents (13-17y)** *N* = 62^1^	**Young Adults (18-25y)** *N* = 28^1^	**p-Value**^**2**^
Fmax Session 1 in [kg]	14 ± 5 (14; 12 - 18); Min: 5, Max: 23 [0]	26 ± 10 (26; 18 - 33); Min: 3, Max: 56 [0]	35 ± 7 (36; 33 - 40); Min: 21, Max: 46 [0]	<0.001***	19.2 ± 4.2 (17.8; 15.0 - 24.4); Min: 14.7, Max: 25.2 [0]	17.9 ± 6.7 (18.9; 13.1 - 22.0); Min: 0.0, Max: 38.5 [0]	17.9 ± 7.1 (18.0; 15.1 - 21.9); Min: 0.0, Max: 35.0 [1]	0.924
Fmean Session 1 in [kg]	12 ± 4 (12; 9 - 14); Min: 3, Max: 20 [0]	21 ± 10 (21; 15 - 28); Min: 2, Max: 47 [0]	31 ± 8 (31; 30 - 37); Min: 15, Max: 42 [0]	<0.001***	16.1 ± 4.4 (15.3; 12.2 - 20.4); Min: 11.6, Max: 23.7 [0]	14.3 ± 6.2 (15.6; 9.1 - 18.8); Min: 0.0, Max: 31.1 [0]	14.5 ± 6.6 (13.8; 11.5 - 17.2); Min: 0.0, Max: 30.6 [1]	0.823
FR Session 1	1.26 ± 0.18 (1.18; 1.15 - 1.32); Min: 1.04, Max: 1.63 [0]	1.26 ± 0.30 (1.18; 1.11 - 1.28); Min: 1.06, Max: 2.42 [0]	1.14 ± 0.10 (1.10; 1.08 - 1.17); Min: 1.08, Max: 1.40 [0]	0.141	1.21 ± 0.08 (1.21; 1.16 - 1.29); Min: 1.07, Max: 1.30 [0]	1.31 ± 0.26 (1.26; 1.14 - 1.39); Min: 1.06, Max: 2.79 [1]	1.28 ± 0.19 (1.23; 1.15 - 1.34); Min: 1.06, Max: 2.01 [2]	0.724
Fmax Session 2 in [kg]	15 ± 3 (14; 12 - 17); Min: 11, Max: 21 [1]	27 ± 11 (27; 18 - 36); Min: 3, Max: 56 [1]	36 ± 7 (38; 34 - 41); Min: 23, Max: 49 [0]	<0.001***	20 ± 4 (21; 15 - 24); Min: 15, Max: 24 [0]	18 ± 8 (19; 13 - 23); Min: 0, Max: 36 [1]	16 ± 7 (16; 11 - 22); Min: 0, Max: 31 [2]	0.355
Fmean Session 2 in [kg]	11 ± 4 (12; 9 - 13); Min: 5, Max: 16 [1]	22 ± 10 (22; 15 - 28); Min: 2, Max: 45 [1]	30 ± 8 (30; 29 - 35); Min: 13, Max: 44 [0]	<0.001***	16.8 ± 4.2 (17.9; 12.7 - 19.9); Min: 11.9, Max: 22.3 [0]	14.4 ± 6.9 (15.1; 8.3 - 18.9); Min: 0.0, Max: 30.3 [1]	12.9 ± 6.4 (13.3; 7.0 - 18.0); Min: 0.0, Max: 26.4 [2]	0.289
FR Session 2	1.48 ± 0.64 (1.27; 1.18 - 1.43); Min: 1.08, Max: 3.21 [1]	1.30 ± 0.29 (1.23; 1.15 - 1.30); Min: 1.06, Max: 2.33 [1]	1.26 ± 0.28 (1.16; 1.12 - 1.27); Min: 1.07, Max: 2.08 [0]	0.332	1.18 ± 0.08 (1.21; 1.09 - 1.21); Min: 1.06, Max: 1.31 [0]	1.30 ± 0.18 (1.26; 1.17 - 1.38); Min: 1.09, Max: 2.00 [2]	1.30 ± 0.22 (1.21; 1.15 - 1.39); Min: 1.10, Max: 2.02 [3]	0.212
Recovery Ratio	1.02 ± 0.26 (1.01; 0.93 - 1.07); Min: 0.56, Max: 1.51 [1]	1.03 ± 0.10 (1.04; 0.97 - 1.08); Min: 0.74, Max: 1.29 [1]	0.95 ± 0.09 (0.95; 0.85 - 1.01); Min: 0.82, Max: 1.12 [0]	0.122	1.05 ± 0.13 (0.98; 0.95 - 1.12); Min: 0.94, Max: 1.29 [0]	0.98 ± 0.19 (0.97; 0.87 - 1.05); Min: 0.49, Max: 1.46 [2]	0.90 ± 0.18 (0.88; 0.80 - 1.06); Min: 0.51, Max: 1.22 [3]	0.072.

^1^ Mean ± SD (Median; Q1 - Q3); Min, Max [# Missing]

^2^ Kruskal-Wallis rank sum test

*p*-values are indicated by *p* < 0.1(.), p < 0.05(*), p < 0.01(**), p < 0.001(***)

Abbreviations: Fmax - maximum force per session; Fmean - mean force per session; FR - fatigue ratio per session

Among male patients, Fmax and Fmean of both sessions differed significantly between age groups (all *p* < 0.001***), while no such differences were observed in female patients. The other HGS indices did not differ significantly across age groups. Sex- and age-stratified analyses of HGS indices, for ME/CFS and noME/CFS patients, are provided in Suppl. Table S3.

In a sensitivity analysis restricting the ME/CFS group to patients with confirmed CCC-ME/CFS and comparing them to noME/CFS disease controls, the overall marginal mean HGS was lower in confirmed CCC-ME/CFS by −3.02 kg (95% CI: −6.42 to 0.38).

### Correlation of handgrip strength with markers of ME/CFS severity

**Fig. 2:**
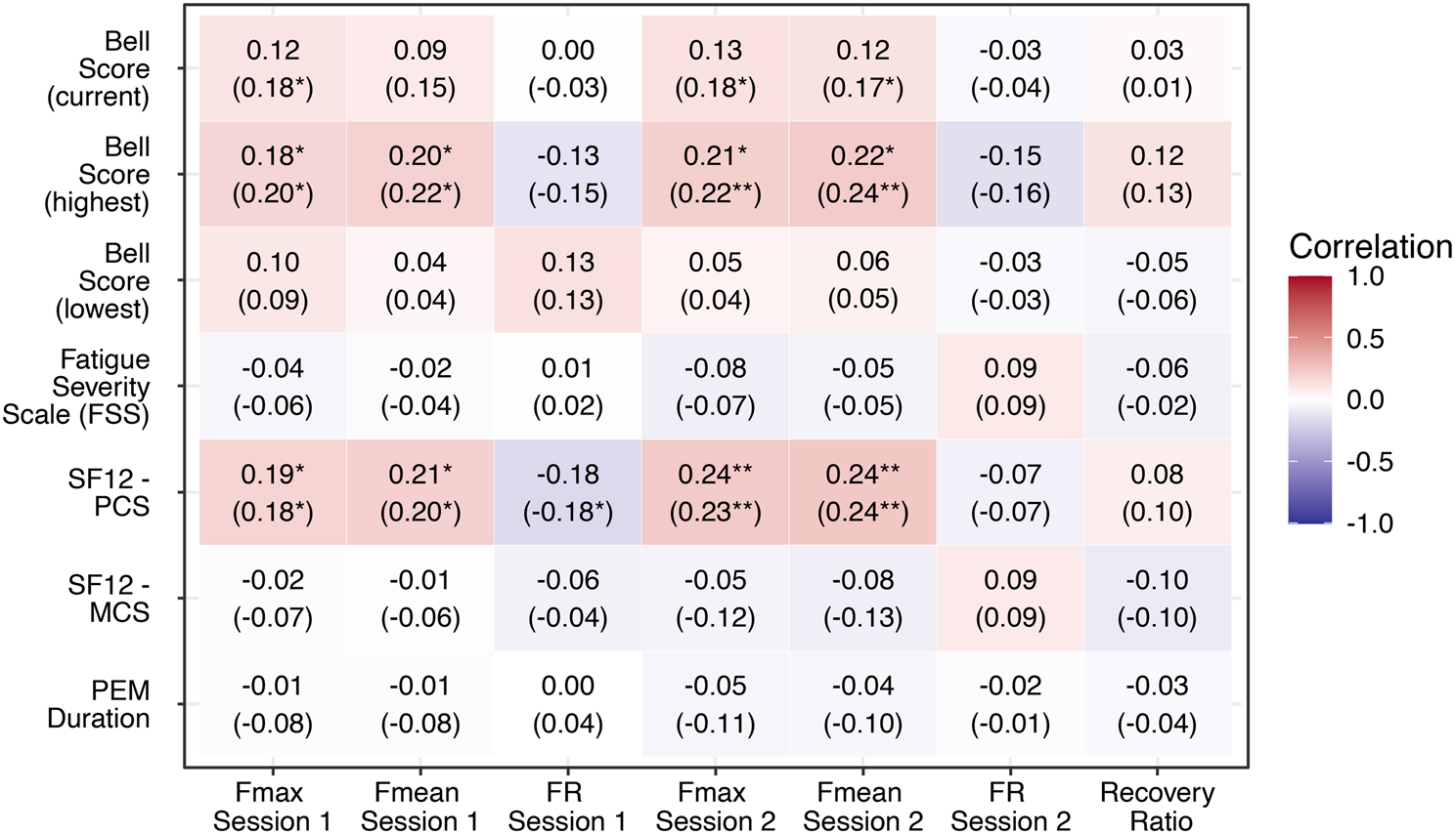
The heatmap shows Spearman partial correlation coefficients between hand grip strength (HGS) parameters and patient-reported outcome measures (PROMs) in the patient group. Partial correlations were adjusted for sex, age, and body mass index (BMI). For each comparison, the adjusted correlation coefficients are displayed, with the raw correlations in parentheses. *p*-values are indicated as follows: *p* < 0.1 (.), *p* < 0.05 (*), *p* < 0.01 (**), *p* < 0.001 (***)

For patients, Fig. 2 shows the raw correlations and partial correlations adjusted for sex, age, and BMI, between the HGS indices and the PROMs (Bell Score, FSS, SF12-PCS, SF12-MCS, and PEM duration).

The highest Bell Score since disease onset and the SF12-PCS significantly correlated with Fmax of sessions 1 and 2, as well as with Fmean of sessions 1 and 2.

**Fig. 3:**
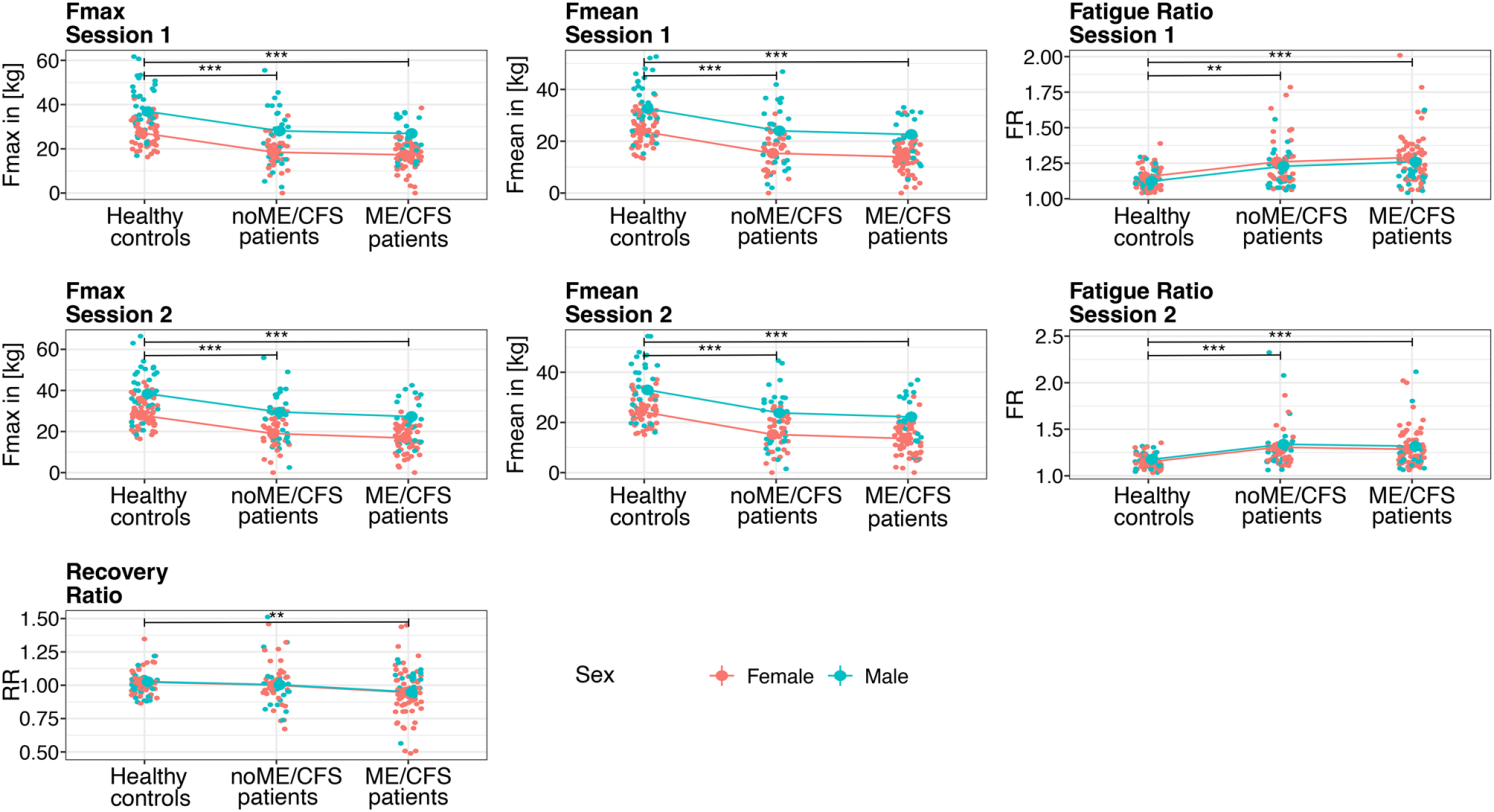
Marginal means of all handgrip strength (HGS) indices across healthy controls, noME/CFS patients, and ME/CFS patients, adjusted for age and body mass index (BMI), for male (green) and female (red) participants. Estimates were obtained from linear models that included group, sex, age, and BMI as independent variables, and each HGS index as the dependent variable. Horizontal lines indicate significant differences between groups. *p*-values represent pairwise comparisons adjusted using Tukey’s method for multiple testing and are indicated as follows: *p* < 0.1 (.), *p* < 0.05 (*), *p* < 0.01 (**), *p* < 0.001 (***). Outliers above the fatigue ratio (FR) cutoffs were excluded from the plot for clarity, but were retained in the statistical analyses. These included, for FR in session 1 (cutoff = 2.0): 0 in healthy controls, 1 in noME/CFS patients, and 3 in ME/CFS patients; and for FR in session 2 (cutoff = 2.5): 0 in healthy controls, 1 in noME/CFS patients, and 0 in ME/CFS patients

Figure 3 compares the HGS parameters between HC, noME/CFS, and ME/CFS patients. Significant differences between the marginal means of HC and noME/CFS patients were observed for Fmax, Fmean, and FR of both sessions. The RR did not significantly differ between HC and noME/CFS patients.

Significant differences between the marginal means of HC and ME/CFS patients were observed for Fmax, Fmean, and FR of both sessions, as well as for the RR. No significant differences were found between noME/CFS and ME/CFS patients for any of the seven HGS parameters. Between noME/CFS and CCC-ME/CFS patients Fmean of session 1 and 2, Fmax of session 2, and the RR significantly differed (Suppl. Fig. S1).

### Diagnostic value of handgrip strength

**Fig. 4:**
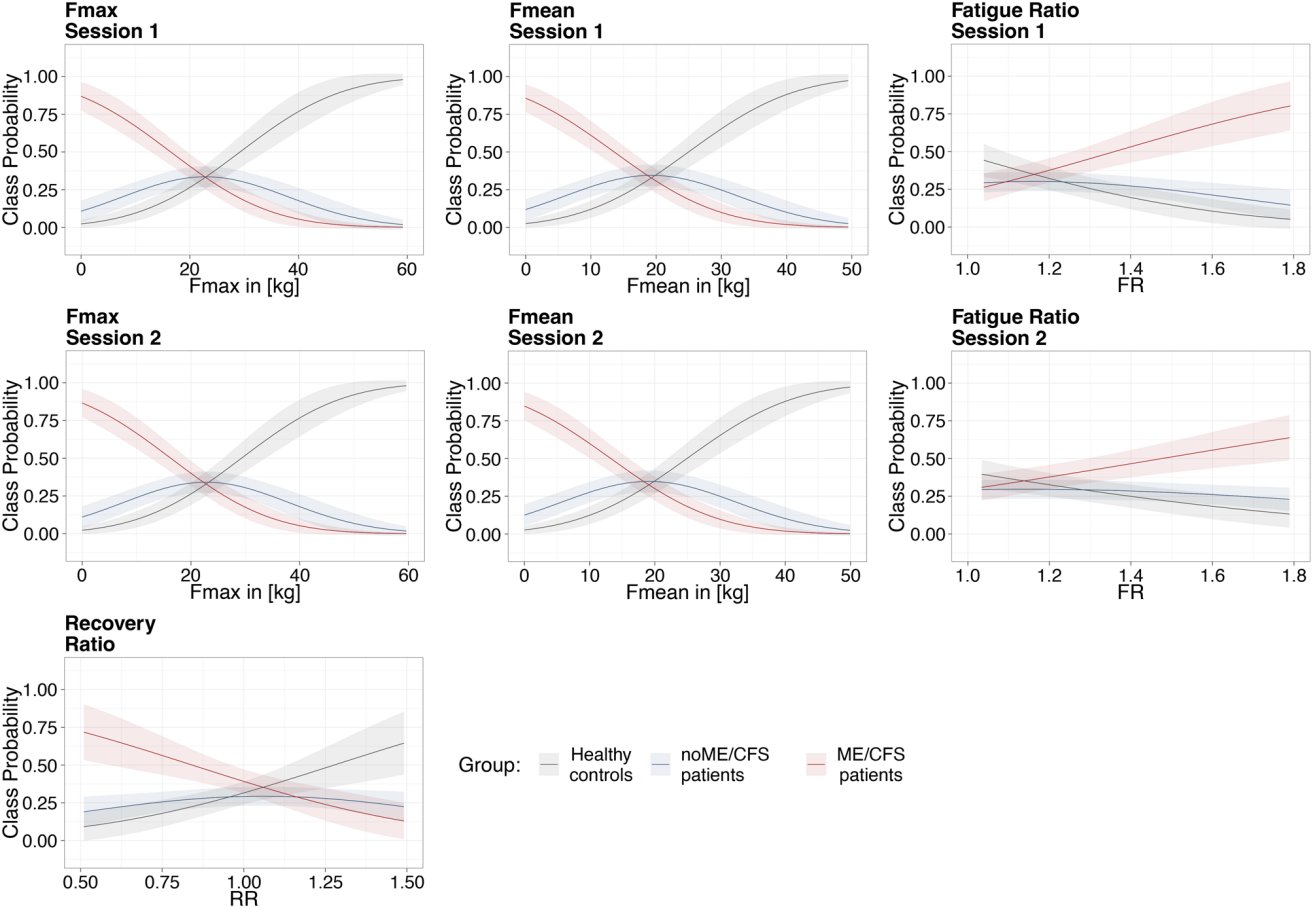
Predicted class probabilities for healthy controls, noME/CFS patients, and ME/CFS patients based on seven handgrip strength (HGS) indices. Results are derived from proportional odds models adjusted for sex, age, and body mass index (BMI)

In proportional odds models (Fig. 4, Table 3) for HC, noME/CFS, and ME/CFS, all seven HGS parameters significantly improved the model fit (all *p* < 0.001***). Accuracies ranged between 49.2% (Fmax of session 2) and 57.3% (Fmean of session 2). All accuracies were significantly greater than the no-information rate of 36.5% (all *p* < 0.001***). Model fits are summarized in Table 3, and by sex and age group in Suppl. Table S4. Figure 4 displays covariate-adjusted class probabilities across HGS indices. Overall, these models showed that each HGS index contains statistically detectable information for distinguishing HC, noME/CFS, and ME/CFS, with moderate overall accuracy.

**Table 3:** Accuracy, C-statistics, and likelihood ratio test (LRT) of proportional odds models assessing the diagnostic value of the seven hand grip strength (HGS) parameters in distinguishing healthy controls, noME/CFS, and ME/CFS patients

Parameters	Accuracy in % (95%-CI)	C-Statistic	**p-Value**^**1**^
Fmax Session 1	55.9 (49.2–62.4)	0.75	<0.001***
Fmean Session 1	56.8 (50.1–63.3)	0.77	<0.001***
FR Session 1	51.1 (44.4–57.8)	0.70	<0.001***
Fmax Session 2	56.0 (49.2–62.6)	0.76	<0.001***
Fmean Session 2	57.3 (50.6–63.9)	0.77	<0.001***
FR Session 2	52.5 (45.7–59.2)	0.70	<0.001***
Recovery Ratio	51.1 (44.4–57.9)	0.68	0.001**

^1^*p*-values are based on likelihood ratio tests comparing the full model to the reduced model. Models were adjusted for sex, age, and BMI

*p*-values are indicated by *p* < 0.1(.), p < 0.05(*), p < 0.01(**), p < 0.001(***)

Abbreviations: CI – Confidence interval; Fmax - maximum force per session in [kg]; Fmean - mean force per session in [kg]; FR - fatigue ratio per session

As a sensitivity analysis, we repeated models comparing noME/CFS patients with confirmed CCC-ME/CFS patients. Discrimination was moderate (accuracy 62.8–70.7%; C-statistic 0.63–0.73), with best performance for absolute HGS strength indices and the RR (Suppl. Table S5). 

Table 4 summarizes sensitivity, specificity, PPV, and NPV for the seven HGS indices across HC, noME/CFS, and ME/CFS. Across parameters, classification performance was highest for HC, consistent with the clear separation of healthy individuals from symptomatic patients.

**Table 4:** Classification metrics of proportional odds models assessing the diagnostic value of the seven handgrip strength parameters in distinguishing healthy controls, noME/CFS, and ME/CFS patients

Parameter	Group	**Sensitivity**^**1**^**(%)**	**Specificity**^**2**^**(%)**	**PPV**^**3**^**(%)**	**NPV**^**4**^**(%)**
Fmax Session 1	HC	77.1	73.3	62.1	84.9
	noME/CFS	1.6	97.6	20.0	72.8
	ME/CFS	75.0	60.0	52.1	80.6
Fmean Session 1	HC	79.5	71.9	61.7	86.1
	noME/CFS	6.5	94.6	30.8	73.1
	ME/CFS	71.4	66.2	55.0	80.0
FR Session 1	HC	77.1	59.7	52.5	81.9
	noME/CFS	0.0	100.0	NA	73.1
	ME/CFS	62.7	63.2	49.5	74.6
Fmax Session 2	HC	80.7	69.7	60.9	86.1
	noME/CFS	6.6	95.1	33.3	73.2
	ME/CFS	67.9	66.7	53.4	78.7
Fmean Session 2	HC	84.3	71.8	63.6	88.7
	noME/CFS	6.6	92.7	25.0	72.7
	ME/CFS	67.9	69.4	55.6	79.4
FR Session 2	HC	78.3	61.4	54.6	82.7
	noME/CFS	0.0	100.0	NA	73.1
	ME/CFS	65.0	63.6	50.0	76.5
Recovery Ratio	HC	69.9	62.1	52.3	77.7
	noME/CFS	0.0	100.0	NA	73.1
	ME/CFS	70.0	60.8	50.0	78.4

Models were adjusted for sex, age, and BMI

^1^ Sensitivity indicates the proportion of true positive cases correctly identified by the model

^2^ Specificity indicates the proportion of true negative cases correctly identified by the model

^3^ PPV indicates the proportion of predicted positive cases that are truly positive

^4^ NPV indicates the proportion of predicted negative cases that are truly negative

Abbreviations: PPV – Positive Predicted Value; NPV – Negative Predicted Value; Fmax - maximum force per session; Fmean - mean force per session; FR - fatigue ratio per session; HC – Healthy control; NA – not available

Among noME/CFS patients, the highest sensitivity was observed for Fmax and Fmean of session 2 (6.6%), while the highest specificity (100.0%) was found across three parameters (FR sessions 1 and 2, and RR). Fmax of session 2 showed the highest PPV (33.3%) and NPV (73.2%). For noME/CFS, sensitivity was uniformly low across parameters, meaning that few noME/CFS patients were correctly classified as noME/CFS by HGS-based models.

In ME/CFS patients, Fmax of session 1 showed the highest sensitivity (75.0%), Fmean of session 2 the highest specificity (69.4%) and PPV (55.6%), and Fmax of session 1 showed the highest NPV (80.6%). For ME/CFS, sensitivity was high for some parameters, but specificity and PPV were moderate, indicating overlap in HGS indices between ME/CFS and noME/CFS patients.

Predictive performance varied by sex and age group (Suppl. Table S6), with higher accuracies/C-statistics in females and young adults and lower performance in males and adolescents. Predictive performance for confirmed CCC-ME/CFS sensitivity analysis stratified by sex and age is shown in Suppl. Table S7.

## Discussion

This prospective observational study, to our knowledge, presents the first data on HGS of CYP with ME/CFS. By the inclusion of HC and disease controls with similar symptoms, we demonstrated i) that a standardized repeated HGS testing protocol in pediatric patients with PEM is clinically feasible, ii) that HGS parameters correlated with global health status and physical functioning but not with PEM duration, and iii) that HGS did not discriminate between PEM-reporting CYP with and without ME/CFS.

### Feasibility of HGS measurement in young people with ME/CFS

Completion rates of the HGS testing protocol were high despite substantial symptom burden. 99.3% patients completed session 1, and 96.6% completed session 2. The protocol confirmed robust differences between distinct groups, with lower HGS in patients compared to HC and in females compared to males, and allowed quantification of within-session fatigability and between-session recovery. These data indicate that a 2-session, 10-trial-per-session protocol can be implemented in routine assessments of CYP with ME/CFS.

Importantly, our protocol addressed aspects that single-burst measurements cannot. Beyond maximal/mean force, both fatigability and recoverability capture strength decline within a session and recovery across sessions [16, 20]. This granularity was not available in brief single-session protocols (e.g., three pulls with long rests [15, 36]) and supports the clinical distinction between low peak strength (weakness) and disproportionate strength decline during repeated trials and/or delayed recovery between sessions [16, 20]. Since FR and RR were inherently protocol-dependent (e.g., number of trials, rests, and session spacing), protocol-specific reference values are required as a consequence, not only for Fmax/Fmean but also for FR and RR.

In this study, the protocol for adults with ME/CFS developed by Jäkel et al. (2021) was adopted for pediatric patients, with the same low-cost digital dynamometer to achieve inter-institutional comparability and scalability despite limited budgets. Absolute HGS values in our young adult subgroup were comparable to those reported by Jäkel et al. (2021), supporting external validity when the same setup was applied. While hydraulic devices are the calibration standard [37, 38], an easily affordable device might be a more suitable option for roll-out in real-world settings. The choice and setup of devices for measurement (handle, posture, rests, feedback) definitely influence absolute handgrip strength [38] and possibly fatigue kinetics. Normal standard values are required for the two-session protocol, particularly for FR and RR.

### Correlation with disease severity

Across patients, HGS reflected the physical disease burden. Maximal and mean force showed modest, consistent positive associations with physical functioning (SF12 PCS) and with the highest Bell score since onset, indicating that higher absolute HGS reflects better physical capacity. Correlations with self-reported PEM duration were absent, and those with mental health (SF12-MCS) were weaker, suggesting that HGS in this age group primarily reflects overall physical impairment and fatigue-related functioning rather than PEM duration or psychosocial features.

This contrasts with adult ME/CFS data that used the same protocol, where PEM measures correlated with specific HGS indices, e.g., lower Fmean in females and higher FR in session 2 in both sexes correlated significantly with higher PEM scores [16], and a lower HGS (Fmax/Fmean) of both sessions correlated significantly with more PEM in female patients [20]. Sex- and age-specific patterns in our younger cohort of patients may have diluted PEM–HGS associations and may partly explain why we did not observe correlations with PEM duration in this pediatric/young adult referral cohort. In the present study, RR differed between HC and ME/CFS patients, but did not distinguish ME/CFS from noME/CFS patients, and did not correlate with PEM duration. RR might rather reflect short-term recovery than delayed PEM-related recovery. Future protocols should include delayed follow-up to distinguish immediate fatigability from PEM-related recovery abnormalities, for example, by combining repeated HGS or other low-burden functional assessments with symptom ratings up to 12, 24, and 48 hours after exertion. In addition, future studies could directly compare patients with chronic fatigue with PEM (ICD-10-GM R53.0) and chronic fatigue without PEM (ICD-10-GM R53.1) to better delineate PEM-specific alterations in recovery dynamics.

Group comparisons supported this pattern: both noME/CFS and ME/CFS patients had substantially lower Fmax/Fmean and altered fatigability compared with HC, with no differences between noME/CFS and ME/CFS, and RR differing only for ME/CFS vs HC. The disease-control group (noME/CFS) included a broad spectrum of diagnoses, including fatigue-related, autonomic, pain-related, sleep-related, neurocognitive, psychological, and other somatic conditions. This clinical heterogeneity likely contributed to the overlap in HGS indices between noME/CFS and ME/CFS patients. Taken together, reduced strength and short-term fatigability may represent non-specific markers of fatigue and exercise intolerance, rather than discriminators of ME/CFS versus noME/CFS. The smaller within-session decline in strength observed in patients, despite their generally lower absolute strength, may reflect pacing strategies, early symptom-related disengagement from the task, or floor effects. Because subjective exertion ratings were not collected, the present data cannot distinguish true muscular fatigability from pacing-related HGS-reduction. Medication use, such as centrally acting drugs, and key symptoms such as orthostatic intolerance or pain may also have influenced HGS performance in both groups. Since these factors were not systematically modeled, they may have introduced residual confounding. This highlights that fatigability metrics depend on baseline strength and testing protocol. Measurement of HGS can thus provide important quantitative indicators of PEM-patients’ general physical status and serve as an objective outcome for monitoring or trials. However, HGS data should be interpreted together with clinical criteria to diagnose ME/CFS, and do not represent a specific biomarker for ME/CFS.

### Diagnostic utility of HGS

Taken together, our models suggest that HGS, as a single diagnostic instrument, has limited utility to distinguish between patients with and without ME/CFS. However, single-parameter proportional-odds models achieved accuracies of 49–57% (all above the 36.5% no-information rate), with clear separation of HC yet substantial overlap between noME/CFS and ME/CFS.

Performance varied by metric. The Fmean of session 2 showed the best sensitivity of HC identification (sensitivity 84.3%) and the highest NPV (88.7%), supporting its use as a rule-out aid in low-pretest-probability settings, i.e., a robust Fmean of session 2 might reduce unnecessary ME/CFS-specific diagnostic work-up in synergy with results from the DSQ-PEM in individuals with mild fatiguing conditions. In turn, Fmax in session 1 showed the highest sensitivity for ME/CFS (75.0%) with an NPV of 80.6%, which may be useful for prioritizing patients with self-reported PEM for comprehensive ME/CFS-specific assessments. However, the ability to identify ME/CFS was limited (best PPV 55.6%; specificity for ME/CFS ≤ 69.4%), and HGS data cannot be used as a standalone diagnostic tool to confirm ME/CFS in patients with self-reported PEM. FR and RR contribute descriptive information on fatigability and recovery, but, on their own, they lack sufficient discriminatory power for disease classification.

Subgroup analyses indicated context-dependent utility: discrimination was strongest in females and young adults (accuracy 63–72%; C-statistic 0.82–0.90), moderate in children, and limited in males and adolescents. HGS was therefore most informative in young adult females, while results in males and minors should be interpreted with caution.

In sum, HGS remains useful as a low-cost objective measure of functional impairment, complementing the assessment of self-reported symptoms. It can support patient stratification for specialized care and for distinct research settings, serve as a single or longitudinal outcome measure, contribute to thorough clinical phenotyping and cluster analyses, and thereby facilitate clinical and translational research approaches to the identification of biomarkers and therapeutic targets.

### Strengths and limitations

This study was well controlled by including both HC as well as disease controls with self-reported chronic fatigue and PEM, reducing spectrum bias and reflecting clinically relevant diagnostic settings. We used a standardized, two-session protocol established by Jäkel et al., and implemented it with the same low-cost digital dynamometer. This approach supports scalability beyond research settings and yields patterns consistent with previously reported adult female cohorts. To enhance comparability, all multivariable analyses were adjusted for sex, age, and BMI. High completion rates minimized attrition bias and strengthened internal validity.

Another strength is the use of two complementary analytic perspectives. The primary models reflected a pragmatic “real-world” referral setting by comparing HC, noME/CFS, and a group of patients with confirmed or probable ME/CFS. The CCC-restricted sensitivity analysis compared only patients with confirmed CCC-ME/CFS and without ME/CFS as cases and controls and was thus more specific. Considering both perspectives improved the interpretability by balancing generalizability (real-world triage) against specificity (strict case definition).

However, this was a single-center referral cohort, which may limit generalizability and introduce a selection bias (e.g., higher pretest probability of ME/CFS). The results were device- and protocol-dependent. Moreover, findings may not transfer to hydraulic devices or alternative testing protocols. The cross-sectional design precludes causal inference, and residual confounding (e.g., pain, medications, motivation/effort, as well as hand size/technique) cannot be ruled out. Finally, the 60-minute inter-session interval may not capture later PEM dynamics, potentially underestimating recovery abnormalities.

## Conclusion

Standardized HGS testing is feasible in CYP with chronic fatigue, self-reported PEM, and suspected ME/CFS. HGS parameters correlated modestly with overall physical status, suggesting general functional impairment rather than ME/CFS-specific pathology. Their role in ME/CFS diagnostics proved limited. However, low Fmax of session 1 and low Fmean of session 2 may help prioritize patients with self-reported PEM for specialized care and research, especially in female CYP and young adults.

## Electronic supplementary material

Below is the link to the electronic supplementary material.

Supplementary Material 1: Supplementary Tables

Supplementary Material 2: Supplementary Figure 1

## Data Availability

The data supporting the conclusions of this article are included within the article and its supplementary material.

## Abbreviations

CCC: Canadian Consensus Criteria
COVID-19: Coronavirus Disease 2019
CYP: Children and Young People
DSQ-PEM: DePaul Symptom Questionnaire assessing PEM
EBV: Epstein-Barr Virus
Fmax: Maximal Handgrip Strength
Fmean: Mean Handgrip Strength
FR: Fatigue Ratio
HC: Healthy Controls
HGS: Handgrip Strength
IOM: Institute of Medicine
IQR: Interquartile Range
MBSQ: Munich Berlin Symptom Questionnaire
MCFC: Munich Chronic Fatigue Center for Young People
ME/CFS: Myalgic Encephalomyelitis / Chronic Fatigue Syndrome
NPV: Negative Predictive Value
PCC: Post-COVID-19 Condition
PEM: Post-Exertional Malaise
PPV: Positive Predictive Value
PROM: Patient-Reported Outcome Measure
PVC: Post-Vaccination Condition
RR: Recovery Ratio
SARS-CoV-2: Severe Acute Respiratory Syndrome Coronavirus Type 2
SD: Standard Deviation
SF12: 12-Item Short Form Health Survey
SF12-MCS: Mental Health Component Summary Scale
SF12-PCS: Physical Component Summary Scale
WHO: World Health Organization
