# Supplementary material for: Handgrip strength in children, adolescents, and young adults with suspected myalgic encephalomyelitis/chronic fatigue syndrome
Source: J Transl Med. 2026 Jul 15;24:947. doi: 10.1186/s12967-026-08654-5 (PMC13393269; doi:10.1186/s12967-026-08654-5)
Supplement: Supplementary file 1 — Supplementary Material 1: Supplementary Tables [file 12967_2026_8654_MOESM1_ESM.docx]

# **SUPPLEMENTARY TABLES**

## **Supplementary Table S1.** *Demographics and Clinical Characteristics of ME/CFS Patients, noME/CFS Patients, and Healthy Controls.*

| **Characteristics** | **Healthy Controls** N = 83^1^ | **NoME/CFS** N = 63^1^ | **ME/CFS**  N = 84^1^ | **P-Val.**^2^ |
| --- | --- | --- | --- | --- |
| Sex | | |  | 0.058 |
| Male | 32 / 83 (39%) [0] | 28 / 63 (44%) [0] | 22 / 84 (26%) [0] |  |
| Female | 51 / 82 (61%) [0] | 35 / 63 (56%) [0] | 62 / 84 (74%) [0] |  |
| Age Group (years) | | |  | 0.006** |
| Children (10-12) | 10 / 83 (12%) [0] | 7 / 63 (11%) [0] | 11 / 84 (13%) [0] |  |
| Adolescents (13-17) | 31 / 83 (37%) [0] | 39 / 63 (62%) [0] | 51 / 84 (61%) [0] |  |
| Young Adults (18-25) | 42 / 83 (51%) [0] | 17 / 63 (27%) [0] | 22 / 84 (26%) [0] |  |
| BMI [kg/m^2^] | 21.1 ± 3.1 (21.2; 18.9 - 23.0); Min: 11.5, Max: 30.1 [0] | 21.1 ± 4.1 (20.3; 18.4 - 23.1); Min: 11.6, Max: 34.6 [0] | 21.0 ± 4.1 (20.3; 18.1 - 22.6); Min: 14.2, Max: 32.9 [0] | 0.583 |
| **Patient-Reported Outcome Measures (PROMs)** | | | | |
| Current Bell Score | 98 ± 4 (100; 100 - 100); Min: 90, Max: 100 [0] | 45 ± 16 (40; 40 - 60); Min: 10, Max: 90 [0] | 36 ± 12 (30; 30 - 40); Min: 10, Max: 70 [0] | <0.001*** |
| Bell Score Highest^3^ | - | 20 ± 12 (20; 10 - 30); Min: 0, Max: 50 [5] | 19 ± 12 (20; 10 - 20); Min: 0, Max: 60 [7] | 0.387 |
| Bell Score Lowest^3^ | - | 54 ± 18 (50; 40 - 70); Min: 20, Max: 90 [5] | 54 ± 19 (50; 40 - 70); Min: 20, Max: 100 [7] | 0.960 |
| SF12 - PCS | 56 ± 3 (56; 55 - 57); Min: 45, Max: 63 [11] | 31 ± 11 (32; 22 - 37); Min: 11, Max: 57 [8] | 24 ± 9 (22; 16 - 30); Min: 11, Max: 53 [10] | <0.001*** |
| SF12 - MCS | 52 ± 6 (54; 49 - 57); Min: 35, Max: 61 [11] | 45 ± 11 (46; 38 - 55); Min: 21, Max: 64 [8] | 47 ± 9 (50; 44 - 53); Min: 24, Max: 63 [10] | <0.001*** |
| Fatigue Severity Scale | 1.91 ± 0.87 (1.67; 1.22 - 2.44); Min: 1.00, Max: 4.56 [45] | 6.21 ± 0.86 (6.44; 6.00 - 6.78); Min: 2.78, Max: 7.00 [0] | 6.45 ± 0.54 (6.56; 6.22 - 6.89); Min: 4.00, Max: 7.00 [0] | <0.001*** |
| DSQ-PEM (PEM Screening) | | | | - |
| negative | 81 / 83 (98%) [0] | 0 / 63 (0%) [0] | 0 / 84 (0%) [0] |  |
| positive | 2 / 83 (2.4%) [0] | 63 / 63 (100%) [0] | 84 / 84 (100%) [0] |  |
| DSQ-PEM (PEM Duration: hours) | | |  | - |
| <1 | 54 / 76 (71%) [7] | 2 / 63 (3.2%) [0] | 0 / 84 (0%) [0] |  |
| 2 - 3 | 20 / 76 (26%) [7] | 14 / 63 (22%) [0] | 2 / 84 (2.4%) [0] |  |
| 4 - 10 | 2 / 76 (2.6%) [7] | 12 / 63 (19%) [0] | 8 / 84 (9.5%) [0] |  |
| 11 - 13 | 0 / 76 (0%) [7] | 10 / 63 (16%) [0] | 5 / 84 (6.0%) [0] |  |
| 14 - 23 | 0 / 76 (0%) [7] | 9 / 63 (14%) [0] | 12 / 84 (14%) [0] |  |
| >24 | 0 / 76 (0%) [7] | 16 / 63 (25%) [0] | 57 / 84 (68%) [0] |  |
| **ME/CFS Diagnostic Criteria (MBSQ)** | | | | |
| CCC fulfilled | 0 / 76 (0%) [7] | 6 / 63 (9.5%) [0] | 52 / 84 (62%) [0] | - |
| IOM Criteria fulfilled | 0 / 76 (0%) [7] | 9 / 63 (14%) [0] | 70 / 84 (83%) [0] | - |
| CDW-R Criteria fulfilled^4^ | 0 / 41 (0%) [0] | 3 / 45 (6.7%) [1] | 40 / 62 (65%) [0] | - |
| PCD-J Criteria fulfilled^4^ | 0 / 41 (0%) [0] | 3 / 45 (6.7%) [1] | 36 / 62 (58%) [0] | - |
| **Diagnoses** | | | | |
| ME/CFS (ICD-10 G93.3) |  |  |  | - |
| excluded | 83 / 83 (100%) [0] | 63 / 63 (100%) [0] | 0 / 84 (0%) [0] |  |
| probable^5^ | 0 / 83 (0%) [0] | 0 / 63 (0%) [0] | 30 / 84 (36%) [0] |  |
| confirmed | 0 / 83 (0%) [0] | 0 / 63 (0%) [0] | 54 / 84 (64%) [0] |  |
| Post-COVID (U09.9!)^56^ | 0 / 83 (0%) [0] | 32 / 63 (51%) [0] | 45 / 84 (54%) [0] | - |
| Post-VAC (U12.9!)^6^ | 0 / 83 (0%) [0] | 4 / 63 (6.3%) [0] | 4 / 84 (4.8%) [0] | - |
| ^1^ n/N (%) [# Missing] or Mean ± SD (Median; Q1 - Q3); Min, Max [# Missing].  ^2^ Pearson’s Chi-squared test; Wilcoxon rank sum test.  ^3^ The highest and lowest Bell Score since disease onset was not assessed for health controls.  ^4^ CDW-R and PCD-J were only assessed in children and adolescents aged less then 18 years. Number of missing values refer to the number of patients aged less than 18 years.  ^5^ Most likely diagnosis after extensive diagnostic work-up.  ^6^ Patients with post-COVID-19 condition (U09.9!) and/or post-COVID-19 vaccination condition (U12.9!).  P-values are indicated by P <0.1(.), P <0.05(*), P <0.01(**), P <0.001(***).  Abbreviations: **BMI** - Body Mass Index; **ME/CFS** - myalgic encephalomyelitis / chronic fatigue syndrome;  **PCS** - post-COVID syndrome; **PVS** - post-vaccination syndrome; **SF12-PCS** - Short Form 12 Health Survey (SF12) Physical Component summary scale; **SF12-MCS** - SF12 Mental Health Component summary scale;  **PEM** - Post-Exertional Malaise; **CCC** - Canadian Consensus Criteria; **IOM** - Institute of Medicine; **CDW-R** - Clinical Diagnostic Worksheet by Rowe PC et al. (2017); **PCD-J** - Pediatric case definition by Jason LA et al. (2011). | | | | |

**Supplementary Table S2.**  *Total of* *confirmed and probable diagnoses (ICD-10-GM) of the noME/CFS group aggregates to a three-character ICD-10-GM Code. Individuals could have more than one diagnosis. Only codes that appeared at least 4 times are included.*

| **ICD-10-GM Code** | **Diagnosis** | **Total Number of Diagnoses** |
| --- | --- | --- |
| R53 | Malaise and fatigue | 46 |
| Z73 | Problems related to life-management difficulty | 42 |
| Z55 | Problems related to education and literacy | 37 |
| G47 | Sleep disorders | 24 |
| F06 | Other mental disorders due to brain damage and dysfunction and to physical disease | 22 |
| G90 | Disorders of autonomic nervous system | 22 |
| E55 | Vitamin D deficiency | 16 |
| F43 | Reaction to severe stress, and adjustment disorders | 16 |
| G44 | Other headache syndromes | 15 |
| Z63 | Other problems related to primary support group, including family circumstances | 15 |
| M79 | Other soft tissue disorders, not elsewhere classified | 14 |
| Z81 | Family history of mental and behavioural disorders | 14 |
| F32 | Depressive episode | 13 |
| F81 | Specific developmental disorders of scholastic skills | 11 |
| R51 | Headache | 10 |
| F40 | Phobic anxiety disorders | 9 |
| G43 | Migraine | 9 |
| R42 | Dizziness and giddiness | 9 |
| F45 | Somatoform disorders | 8 |
| I95 | Hypotension | 7 |
| R00 | Abnormalities of heart beat | 7 |
| F93 | Emotional disorders with onset specific to childhood | 6 |
| H93 | Other disorders of ear, not elsewhere classified | 6 |
| E66 | Obesity | 5 |
| J45 | Asthma | 5 |
| M25 | Other joint disorders, not elsewhere classified | 5 |
| R06 | Abnormalities of breathing | 5 |
| Z82 | Family history of certain disabilities and chronic diseases leading to disablement | 5 |
| F90 | Hyperkinetic disorders | 4 |
| R10 | Abdominal and pelvic pain | 4 |

## **Supplementary Table S3.** *Handgrip Strength in NoME/CFS (A) and ME/CFS (B) Patients Stratified by Sex and Age.*

| **A) NoME/CFS Patients** | | | | | | | | |
| --- | --- | --- | --- | --- | --- | --- | --- | --- |
| Parameters | **Male** | | | | **Female** | | | |
|  | **Children (10-12y)**  N = 6^1^ | **Adolescents (13-17y)**  N = 14^1^ | **Young Adults (18-25y)**  N = 8^1^ | **P-Value^2^** | **Children  (10-12y)**  N = 1^1^ | **Adolescents (13-17y)**  N = 25^1^ | **Young Adults (18-25y)**  N = 9^1^ | **P-Value^2^** |
| Fmax Session 1 in [kg] | 13 ± 6 (13; 9 - 18); Min: 5, Max: 23 [0] | 25 ± 12 (26; 16 - 30); Min: 3, Max: 56 [0] | 37 ± 8 (40; 35 - 41); Min: 21, Max: 46 [0] | **0.001**** | 15.0 ± NA (15.0; 15.0 – 15.0); Min: 15.0, Max: 15.0 [0] | 18.3 ± 6.7 (20.2; 14.7 – 22.2); Min: 0.0, Max: 28.2 [0] | 21.8 ± 7.6 (20.6; 19.3 – 25.4); Min: 8.9, Max: 35.0 [1] | 0.460 |
| Fmean Session 1 in [kg] | 11 ± 6 (11; 7 – 14); Min: 3, Max: 20 [0] | 21 ± 11 (22; 13 – 27); Min: 2, Max: 47 [0] | 33 ± 8 (36; 31 – 37); Min: 15, Max: 42 [0] | **0.001**** | 11.6 ± NA (11.6; 11.6 - 11.6); Min: 11.6, Max: 11.6 [0] | 14.9 ± 6.6 (17.7; 8.8 - 19.1); Min: 0.0, Max: 26.4 [0] | 18.5 ± 7.2 (17.0; 15.4 - 23.1); Min: 6.4, Max: 30.6 [1] | 0.514 |
| FR Session 1 | 1.28 ± 0.16 (1.27; 1.15 - 1.32); Min: 1.12, Max: 1.56 [0] | 1.24 ± 0.26 (1.16; 1.09 - 1.30); Min: 1.06, Max: 2.07 [0] | 1.15 ± 0.11 (1.09; 1.08 - 1.19); Min: 1.08, Max: 1.40 [0] | 0.170 | 1.29 ± NA (1.29; 1.29 - 1.29); Min: 1.29, Max: 1.29 [0] | 1.29 ± 0.21 (1.19; 1.15 - 1.44); Min: 1.07, Max: 1.79 [1] | 1.21 ± 0.11 (1.19; 1.14 - 1.28); Min: 1.06, Max: 1.40 [1] | 0.520 |
| Fmax Session 2 in [kg] | 16 ± 4 (15; 12 - 19); Min: 12, Max: 21 [0] | 26 ± 13 (28; 17 - 30); Min: 3, Max: 56 [1] | 38 ± 7 (39; 35 - 41); Min: 26, Max: 49 [0] | **0.002**** | 15 ± NA (15; 15 – 15); Min: 15, Max: 15 [0] | 19 ± 8 (22; 14 – 26); Min: 0, Max: 31 [0] | 21 ± 8 (22; 18 – 27); Min: 6, Max: 31 [1] | 0.662 |
| Fmean Session 2 in [kg] | 11 ± 4 (11; 9 – 13); Min: 5, Max: 16 [0] | 21 ± 11 (24; 13 – 28); Min: 2, Max: 45 [1] | 31 ± 9 (30; 29 – 36); Min: 13, Max: 44 [0] | **0.003**** | 13 ± NA (13; 13 - 13); Min: 13, Max: 13 [0] | 16 ± 7 (18; 9 - 21); Min: 0, Max: 26 [0] | 18 ± 6 (18; 15 - 21); Min: 5, Max: 26 [1] | 0.748 |
| FR Session 2 | 1.61 ± 0.79 (1.29; 1.26 - 1.43); Min: 1.18, Max: 3.21 [0] | 1.33 ± 0.34 (1.21; 1.15 - 1.33); Min: 1.06, Max: 2.33 [1] | 1.30 ± 0.33 (1.19; 1.14 - 1.31); Min: 1.07, Max: 2.08 [0] | 0.298 | 1.21 ± NA (1.21; 1.21 - 1.21); Min: 1.21, Max: 1.21 [0] | 1.29 ± 0.19 (1.22; 1.16 - 1.33); Min: 1.10, Max: 1.86 [1] | 1.21 ± 0.06 (1.20; 1.17 - 1.25); Min: 1.15, Max: 1.33 [1] | 0.897 |
| Recovery Ratio | 1.10 ± 0.27 (1.01; 0.93 - 1.32); Min: 0.80, Max: 1.51 [0] | 1.01 ± 0.12 (1.01; 0.95 - 1.06); Min: 0.74, Max: 1.29 [1] | 0.93 ± 0.08 (0.95; 0.85 - 0.99); Min: 0.82, Max: 1.04 [0] | 0.215 | 1.10 ± NA (1.10; 1.10 - 1.10); Min: 1.10, Max: 1.10 [0] | 1.03 ± 0.18 (1.01; 0.94 - 1.10); Min: 0.67, Max: 1.46 [1] | 0.96 ± 0.15 (0.99; 0.85 - 1.06); Min: 0.73, Max: 1.18 [1] | 0.498 |
| **B) ME/CFS Patients** | | | | | | | | |
| **Parameters** | **Male** | | | | **Female** | | | |
|  | **Children (10-12y)**  N = 5^1^ | **Adolescents (13-17y)**  N = 14^1^ | **Young Adults (18-25y)**  N = 3^1^ | **P-Value^2^** | **Children (10-12y)**  N = 6^1^ | **Adolescents (13-17y)**  N = 37^1^ | **Young Adults (18-25y)**  N = 19^1^ | **P-Value^2^** |
| Fmax Session 1 in [kg] | 15 ± 4 (14; 14 - 16); Min: 12, Max: 22 [0] | 26 ± 8 (26; 20 - 34); Min: 13, Max: 37 [0] | 31 ± 5 (34; 25 - 34); Min: 25, Max: 34 [0] | **0.021*** | 19.9 ± 4.1 (18.9; 17.6 - 24.4); Min: 14.7, Max: 25.2 [0] | 17.6 ± 6.8 (18.7; 12.7 - 21.5); Min: 2.8, Max: 38.5 [0] | 16.2 ± 6.4 (17.0; 13.8 - 19.4); Min: 0.0, Max: 25.7 [0] | 0.433 |
| Fmean Session 1 in [kg] | 13 ± 1 (13; 12 - 14); Min: 10, Max: 14 [0] | 22 ± 8 (20; 17 - 30); Min: 5, Max: 33 [0] | 28 ± 6 (30; 21 - 31); Min: 21, Max: 31 [0] | 0.017* | 16.9 ± 4.3 (15.7; 13.6 - 20.4); Min: 12.2, Max: 23.7 [0] | 14.0 ± 6.0 (15.5; 10.5 - 18.0); Min: 2.1, Max: 31.1 [0] | 12.8 ± 5.6 (12.5; 11.5 - 16.1); Min: 0.0, Max: 22.2 [0] | 0.350 |
| FR Session 1 | 1.23 ± 0.23 (1.16; 1.15 - 1.18); Min: 1.04, Max: 1.63 [0] | 1.27 ± 0.34 (1.18; 1.14 - 1.25); Min: 1.06, Max: 2.42 [0] | 1.12 ± 0.04 (1.12; 1.09 - 1.17); Min: 1.09, Max: 1.17 [0] | 0.454 | 1.20 ± 0.08 (1.20; 1.16 - 1.24); Min: 1.07, Max: 1.30 [0] | 1.32 ± 0.30 (1.27; 1.14 - 1.37); Min: 1.06, Max: 2.79 [0] | 1.30 ± 0.22 (1.25; 1.16 - 1.37); Min: 1.06, Max: 2.01 [1] | 0.412 |
| Fmax Session 2 in [kg] | 14 ± 2 (14; 12 - 15); Min: 11, Max: 16 [1] | 28 ± 10 (26; 22 - 37); Min: 10, Max: 43 [0] | 32 ± 8 (34; 23 - 38); Min: 23, Max: 38 [0] | 0.039* | 20.3 ± 3.8 (21.2; 17.0 - 23.7); Min: 14.5, Max: 24.1 [0] | 17.3 ± 7.3 (18.4; 12.1 - 22.5); Min: 2.6, Max: 36.2 [1] | 13.8 ± 6.0 (14.7; 9.2 - 17.3); Min: 0.0, Max: 23.4 [1] | 0.065. |
| Fmean Session 2 in [kg] | 11 ± 4 (13; 9 - 13); Min: 6, Max: 14 [1] | 23 ± 9 (22; 18 - 31); Min: 5, Max: 37 [0] | 28 ± 8 (30; 20 - 35); Min: 20, Max: 35 [0] | 0.044* | 17.5 ± 4.1 (18.8; 13.0 - 19.9); Min: 11.9, Max: 22.3 [0] | 13.6 ± 6.5 (15.0; 8.1 - 17.9); Min: 1.8, Max: 30.3 [1] | 10.8 ± 5.3 (10.6; 6.5 - 13.6); Min: 0.0, Max: 19.4 [1] | 0.033* |
| FR Session 2 | 1.29 ± 0.34 (1.15; 1.10 - 1.49); Min: 1.08, Max: 1.80 [1] | 1.28 ± 0.25 (1.24; 1.17 - 1.28); Min: 1.06, Max: 2.12 [0] | 1.13 ± 0.04 (1.15; 1.08 - 1.16); Min: 1.08, Max: 1.16 [0] | 0.157 | 1.17 ± 0.09 (1.19; 1.09 - 1.21); Min: 1.06, Max: 1.31 [0] | 1.31 ± 0.18 (1.29; 1.18 - 1.41); Min: 1.09, Max: 2.00 [1] | 1.34 ± 0.26 (1.21; 1.15 - 1.47); Min: 1.10, Max: 2.02 [2] | 0.170 |
| Recovery Ratio | 0.90 ± 0.23 (0.99; 0.75 - 1.05); Min: 0.56, Max: 1.07 [1] | 1.04 ± 0.08 (1.06; 0.98 - 1.09); Min: 0.91, Max: 1.19 [0] | 1.02 ± 0.09 (0.98; 0.95 - 1.12); Min: 0.95, Max: 1.12 [0] | 0.378 | 1.04 ± 0.14 (0.98; 0.95 - 1.12); Min: 0.94, Max: 1.29 [0] | 0.95 ± 0.19 (0.93; 0.86 - 1.03); Min: 0.49, Max: 1.45 [1] | 0.87 ± 0.20 (0.88; 0.80 - 0.95); Min: 0.51, Max: 1.22 [2] | 0.076. |
| ^1^ Mean ± SD (Median; Q1 - Q3); Min: minimum, Max: maximum [N Missing].  ^2^ Kruskal-Wallis rank sum test.  P-values are indicated by P <0.1(.), P < 0.05 (*), P < 0.01 (**), P < 0.001 (***).  Abbreviations: **Fmax** - maximum strength per session; **Fmean** - mean strength per session; **FR** - fatigue ratio per session. | | | | | | | | |

## **Supplementary Table S4.** *Performance metrics of proportional odds models assessing the ME/CFS diagnostic value of the seven hand grip strength (HGS) indices in distinguishing healthy controls, noME/CFS, and ME/CFS patients, stratified by sex and age.*

| **Parameters** | **Accuracy %**  **(95%-CI)** | **C-Statistic** | **P-Value^1^** |
| --- | --- | --- | --- |
| **Female**  (N = 148) | | | |
| Fmax Session 1 in [kg] | 61.2 (52.8 – 69.1) | 0.78 | <0.001*** |
| Fmean Session 1 in [kg] | 63.3 (54.9 – 71.1) | 0.79 | <0.001*** |
| FR Session 1 | 52.4 (44.0 – 60.8) | 0.73 | <0.001*** |
| Fmax Session 2 in [kg] | 59.3 (50.8 – 67.4) | 0.80 | <0.001*** |
| Fmean Session 2 in [kg] | 63.4 (55.1 – 71.3) | 0.82 | <0.001*** |
| FR Session 2 | 60.8 (52.3 – 68.9) | 0.76 | <0.001*** |
| Recovery Ratio | 58.7 (50.2 – 66.9) | 0.70 | <0.001*** |
| **Male**  (N = 82) | | | |
| Fmax Session 1 in [kg] | 45.1 (34.1 – 56.5) | 0.74 | <0.001*** |
| Fmean Session 1 in [kg] | 47.6 (36.4 – 58.9) | 0.74 | <0.001*** |
| FR Session 1 | 46.3 (35.3 – 57.7) | 0.64 | 0.182 |
| Fmax Session 2 in [kg] | 47.5 (36.2 – 59.0) | 0.71 | <0.001*** |
| Fmean Session 2 in [kg] | 48.8 (37.4 – 60.2) | 0.71 | <0.001*** |
| FR Session 2 | 45.0 (33.8 – 56.5) | 0.62 | 0.432 |
| Recovery Ratio | 41.2 (30.4 – 52.8) | 0.62 | 0.757 |
| **Children (10-12y)**  (N = 28) | | | |
| Fmax Session 1 in [kg] | 64.3 (44.1 – 81.4) | 0.82 | 0.001** |
| Fmean Session 1 in [kg] | 64.3 (44.1 – 81.4) | 0.79 | 0.002** |
| FR Session 1 | 42.9 (24.5 – 62.8) | 0.65 | 0.748 |
| Fmax Session 2 in [kg] | 63.0 (42.4 – 80.6) | 0.85 | <0.001*** |
| Fmean Session 2 in [kg] | 63.0 (42.4 – 80.6) | 0.80 | 0.001* |
| FR Session 2 | 40.7 (22.4 – 61.2) | 0.63 | 0.673 |
| Recovery Ratio | 40.7 (22.4 – 61.2) | 0.63 | 0.792 |
| **Adolescents (13-17y)**  (N = 121) | | | |
| Fmax Session 1 in [kg] | 48.8 (39.6 – 58.0) | 0.72 | <0.001*** |
| Fmean Session 1 in [kg] | 52.1 (42.8 – 61.2) | 0.73 | <0.001*** |
| FR Session 1 | 49.2 (39.9 – 58.4) | 0.67 | 0.015** |
| Fmax Session 2 in [kg] | 47.9 (38.7 – 57.2) | 0.72 | <0.001*** |
| Fmean Session 2 in [kg] | 48.7 (39.5 – 58.1) | 0.73 | <0.001*** |
| FR Session 2 | 45.8 (36.6 – 55.2) | 0.68 | 0.006** |
| Recovery Ratio | 39.0 (30.1 – 48.4) | 0.62 | 0.103 |
| **Young Adults (18-25y)**  (N = 81) | | | |
| Fmax Session 1 in [kg] | 70.0 (58.7 – 79.7) | 0.86 | <0.001*** |
| Fmean Session 1 in [kg] | 71.3 (60.0 – 80.8) | 0.88 | <0.001*** |
| FR Session 1 | 68.4 (56.9 – 78.4) | 0.80 | <0.001*** |
| Fmax Session 2 in [kg] | 72.2 (60.9 – 81.7) | 0.90 | <0.001*** |
| Fmean Session 2 in [kg] | 74.7 (63.6 – 83.8) | 0.89 | <0.001*** |
| FR Session 2 | 65.4 (53.8 – 75.8) | 0.72 | 0.002** |
| Recovery Ratio | 64.1 (52.4 – 74.7) | 0.73 | <0.001*** |
| ^1^ P-values are based on likelihood ratio tests comparing the full model to the reduced model.  Models were adjusted for sex, age, and BMI.  P-values are indicated by P < 0.1 (.), P < 0.05 (*), P < 0.01 (**), P < 0.001 (***). | | | |

## **Supplementary Table S5.** *Accuracy, C-statistics, and likelihood ratio test (LRT) of proportional odds models assessing the diagnostic value of the seven hand grip strength (HGS) indices in distinguishing noME/CFS from strictly CCC-confirmed ME/CFS patients.*

| **Parameters** | **Accuracy in %  (95%-CI)** | **C-Statistic** | **P-Val.^1^** |
| --- | --- | --- | --- |
| **All Patients**  (N = 103) | | | |
| Fmax Session 1 | 65.7 (55.6 – 74.8) | 0.71 | 0.066 |
| Fmean Session 1 | 64.7 (54.6 – 73.9) | 0.72 | 0.030 |
| FR Session 1 | 65.3 (55.2 – 74.5) | 0.71 | 0.077 |
| Fmax Session 2 | 70.0 (60.0 – 78.8) | 0.73 | 0.007 |
| Fmean Session 2 | 67.0 (56.9 – 76.1) | 0.73 | 0.010 |
| FR Session 2 | 64.6 (54.4 – 74.0) | 0.68 | 0.476 |
| Recovery Ratio | 70.7 (60.7 – 79.4) | 0.72 | 0.013 |
| **Female**  (N = 65) | | | |
| Fmax Session 1 | 60.9 (47.9 – 72.9) | 0.72 | 0.066 |
| Fmean Session 1 | 62.5 (49.5 – 74.3) | 0.73 | 0.031 |
| FR Session 1 | 63.5 (50.4 – 75.3) | 0.70 | 0.103 |
| Fmax Session 2 | 69.8 (57.0 – 80.8) | 0.78 | 0.004 |
| Fmean Session 2 | 66.7 (53.7 – 78.0) | 0.76 | 0.003 |
| FR Session 2 | 62.9 (49.7 – 74.8) | 0.68 | 0.152 |
| Recovery Ratio | 67.7 (54.7 – 79.1) | 0.74 | 0.012 |
| **Male**  (N = 38) | | | |
| Fmax Session 1 | 73.7 (56.9 – 86.6) | 0.65 | 0.895 |
| Fmean Session 1 | 73.7 (56.9 – 86.6) | 0.63 | 0.751 |
| FR Session 1 | 76.3 (59.8 – 88.6) | 0.65 | 0.495 |
| Fmax Session 2 | 73.0 (55.9 – 86.2) | 0.63 | 0.813 |
| Fmean Session 2 | 73.0 (55.9 – 86.2) | 0.63 | 0.937 |
| FR Session 2 | 73.0 (55.9 – 86.2) | 0.64 | 0.836 |
| Recovery Ratio | 73.0 (55.9 – 86.2) | 0.65 | 0.664 |
| **Children (10-12y)**  (N = 9) | | | |
| Fmax Session 1 | 100.0 (66.4 – 100.0) | 1.00 | 0.025 |
| Fmean Session 1 | 100.0 (66.4 – 100.0) | 1.00 | 0.025 |
| FR Session 1 | 88.9 (51.8 – 99.7) | 0.86 | 0.585 |
| Fmax Session 2 | 100.0 (66.4 – 100.0) | 1.00 | 0.025 |
| Fmean Session 2 | 100.0 (66.4 – 100.0) | 1.00 | 0.025 |
| FR Session 2 | 100.0 (66.4 – 100.0) | 1.00 | 0.025 |
| Recovery Ratio | 100.0 (66.4 – 100.0) | 1.00 | 0.025 |
| **Adolescents (13-17y)**  (N = 63) | | | |
| Fmax Session 1 | 65.1 (52.0 – 76.7) | 0.65 | 0.320 |
| Fmean Session 1 | 65.1 (52.0 – 76.7) | 0.67 | 0.214 |
| FR Session 1 | 64.5 (51.3 – 76.3) | 0.68 | 0.170 |
| Fmax Session 2 | 64.5 (51.3 – 76.3) | 0.68 | 0.127 |
| Fmean Session 2 | 64.5 (51.3 – 76.3) | 0.69 | 0.108 |
| FR Session 2 | 63.9 (50.6 – 75.8) | 0.65 | 0.362 |
| Recovery Ratio | 65.6 (52.3 – 77.3) | 0.69 | 0.092 |
| **Young Adults (18-25y)**  (N = 31) | | | |
| Fmax Session 1 | 76.7 (57.7 – 90.1) | 0.90 | 0.040 |
| Fmean Session 1 | 80.0 (61.4 – 92.3) | 0.91 | 0.041 |
| FR Session 1 | 83.3 (65.3 – 94.4) | 0.88 | 0.285 |
| Fmax Session 2 | 89.7 (72.6 – 97.8) | 0.95 | 0.002 |
| Fmean Session 2 | 82.8 (64.2 – 94.2) | 0.92 | 0.013 |
| FR Session 2 | 79.3 (60.3 – 92.0) | 0.88 | 0.788 |
| Recovery Ratio | 75.9 (56.5 – 89.7) | 0.88 | 0.099 |
| ^1^ P-values are based on likelihood ratio tests comparing the full model to the reduced model. Models were adjusted for sex, age, and BMI.  P-values are indicated by P<0.1(.), **P<0.05(*)**, **P<0.01(**)**, **P<0.001(***).**  Abbreviations: **CI** – Confidence interval; **Fmax** - maximum force per session in [kg]; **Fmean** - mean force per session in [kg]; **FR** - fatigue ratio per session. | | | |

## **Supplementary Table S6.** *Classification metrics of Proportional Odds Models assessing the diagnostic of nine handgrip strength (HGS) indices in distinguishing healthy controls, noME/CFS and ME/CFS patients, stratified by sex and age.*

| **Parameter** | **Group** | **Sensitivity^1^ (%)** | **Specificity^2^**  **(%)** | **PPV^3^**  **(%)** | **NPV^4^**  **(%)** |
| --- | --- | --- | --- | --- | --- |
| **Female**  (N = 148) | | | | | |
| Fmax Session 1 | Healthy control | 78.4 | 78.1 | 65.6 | 87.2 |
|  | noME/CFS patient | 0.0 | 100.0 | - | 76.9 |
|  | ME/CFS patient | 80.6 | 57.6 | 58.1 | 80.3 |
| Fmean Session 1 | Healthy control | 82.4 | 79.2 | 67.7 | 89.4 |
|  | noME/CFS patient | 0.0 | 100.0 | - | 76.9 |
|  | ME/CFS patient | 82.3 | 60.0 | 60.0 | 82.3 |
| FR Session 1 | Healthy control | 68.6 | 69.1 | 54.7 | 80.2 |
|  | noME/CFS patient | 0.0 | 100.0 | - | 77.2 |
|  | ME/CFS patient | 67.2 | 52.4 | 50.6 | 68.8 |
| Fmax Session 2 | Healthy control | 80.4 | 69.1 | 58.6 | 86.7 |
|  | noME/CFS patient | 0.0 | 100.0 | - | 76.6 |
|  | ME/CFS patient | 75.0 | 64.7 | 60.0 | 78.6 |
| Fmean Session 2 | Healthy control | 80.4 | 77.7 | 66.1 | 88.0 |
|  | noME/CFS patient | 5.9 | 96.4 | 33.3 | 77.0 |
|  | ME/CFS patient | 81.7 | 67.1 | 63.6 | 83.8 |
| FR Session 2 | Healthy control | 80.4 | 70.7 | 60.3 | 86.7 |
|  | noME/CFS patient | 0.0 | 100.0 | - | 76.9 |
|  | ME/CFS patient | 78.0 | 65.5 | 61.3 | 80.9 |
| Recovery Ratio | Healthy control | 72.5 | 73.9 | 60.7 | 82.9 |
|  | noME/CFS patient | 0.0 | 100.0 | - | 76.9 |
|  | ME/CFS patient | 79.7 | 58.3 | 57.3 | 80.3 |
| **Male**  (N = 82) | | | | | |
| Fmax Session 1 | Healthy control | 68.8 | 74.0 | 62.9 | 78.7 |
|  | noME/CFS patient | 25.0 | 61.1 | 25.0 | 61.1 |
|  | ME/CFS patient | 36.4 | 81.7 | 42.1 | 77.8 |
| Fmean Session 1 | Healthy control | 71.9 | 74.0 | 63.9 | 80.4 |
|  | noME/CFS patient | 28.6 | 64.8 | 29.6 | 63.6 |
|  | ME/CFS patient | 36.4 | 81.7 | 42.1 | 77.8 |
| FR Session 1 | Healthy control | 75.0 | 54.0 | 51.1 | 77.1 |
|  | noME/CFS patient | 32.1 | 77.8 | 42.9 | 68.9 |
|  | ME/CFS patient | 22.7 | 85.0 | 35.7 | 75.0 |
| Fmax Session 2 | Healthy control | 75.0 | 66.7 | 60.0 | 80.0 |
|  | noME/CFS patient | 22.2 | 73.6 | 30.0 | 65.0 |
|  | ME/CFS patient | 38.1 | 79.7 | 40.0 | 78.3 |
| Fmean Session 2 | Healthy control | 75.0 | 64.6 | 58.5 | 79.5 |
|  | noME/CFS patient | 25.9 | 73.6 | 33.3 | 66.1 |
|  | ME/CFS patient | 38.1 | 83.1 | 44.4 | 79.0 |
| FR Session 2 | Healthy control | 75.0 | 52.1 | 51.1 | 75.8 |
|  | noME/CFS patient | 29.6 | 79.2 | 42.1 | 68.9 |
|  | ME/CFS patient | 19.0 | 83.1 | 28.6 | 74.2 |
| Recovery Ratio | Healthy control | 68.8 | 50.0 | 47.8 | 70.6 |
|  | noME/CFS patient | 29.6 | 73.6 | 36.4 | 67.2 |
|  | ME/CFS patient | 14.3 | 84.7 | 25.0 | 73.5 |
| **Children (10-12y)**  (N = 28) | | | | | |
| Fmax Session 1 | Healthy control | 90.0 | 88.9 | 81.8 | 94.1 |
|  | noME/CFS patient | 14.3 | 90.5 | 33.3 | 76.0 |
|  | ME/CFS patient | 72.7 | 64.7 | 57.1 | 78.6 |
| Fmean Session 1 | Healthy control | 90.0 | 77.8 | 69.2 | 93.3 |
|  | noME/CFS patient | 0.0 | 100.0 | - | 75.0 |
|  | ME/CFS patient | 81.8 | 64.7 | 60.0 | 84.6 |
| FR Session 1 | Healthy control | 60.0 | 55.6 | 42.9 | 71.4 |
|  | noME/CFS patient | 0.0 | 100.0 | - | 75.0 |
|  | ME/CFS patient | 54.5 | 52.9 | 42.9 | 64.3 |
| Fmax Session 2 | Healthy control | 90.0 | 88.2 | 81.8 | 93.8 |
|  | noME/CFS patient | 28.6 | 85.0 | 40.0 | 77.3 |
|  | ME/CFS patient | 60.0 | 70.6 | 54.5 | 75.0 |
| Fmean Session 2 | Healthy control | 90.0 | 82.4 | 75.0 | 93.3 |
|  | noME/CFS patient | 28.6 | 90.0 | 50.0 | 78.3 |
|  | ME/CFS patient | 60.0 | 70.6 | 54.5 | 75.0 |
| FR Session 2 | Healthy control | 70.0 | 47.1 | 43.8 | 72.7 |
|  | noME/CFS patient | 0.0 | 100.0 | - | 74.1 |
|  | ME/CFS patient | 40.0 | 58.8 | 36.4 | 62.5 |
| Recovery Ratio | Healthy control | 60.0 | 52.9 | 42.9 | 69.2 |
|  | noME/CFS patient | 0.0 | 100.0 | - | 74.1 |
|  | ME/CFS patient | 50.0 | 52.9 | 38.5 | 64.3 |
| **Adolescents (13-17y)**  (N = 121) | | | | | |
| Fmax Session 1 | Healthy control | 45.2 | 90.0 | 60.9 | 82.7 |
|  | noME/CFS patient | 25.6 | 72.0 | 30.3 | 67.0 |
|  | ME/CFS patient | 68.6 | 57.1 | 53.8 | 71.4 |
| Fmean Session 1 | Healthy control | 51.6 | 90.0 | 64.0 | 84.4 |
|  | noME/CFS patient | 30.8 | 72.0 | 34.3 | 68.6 |
|  | ME/CFS patient | 68.6 | 62.9 | 57.4 | 73.3 |
| FR Session 1 | Healthy control | 32.3 | 85.4 | 43.5 | 78.4 |
|  | noME/CFS patient | 15.8 | 91.5 | 46.2 | 70.1 |
|  | ME/CFS patient | 84.3 | 40.6 | 51.2 | 77.8 |
| Fmax Session 2 | Healthy control | 45.2 | 89.8 | 60.9 | 82.3 |
|  | noME/CFS patient | 26.3 | 70.4 | 29.4 | 67.1 |
|  | ME/CFS patient | 66.0 | 58.0 | 53.2 | 70.2 |
| Fmean Session 2 | Healthy control | 45.2 | 90.9 | 63.6 | 82.5 |
|  | noME/CFS patient | 28.9 | 67.9 | 29.7 | 67.1 |
|  | ME/CFS patient | 66.0 | 60.9 | 55.0 | 71.2 |
| FR Session 2 | Healthy control | 32.3 | 85.1 | 43.5 | 77.9 |
|  | noME/CFS patient | 16.2 | 82.7 | 30.0 | 68.4 |
|  | ME/CFS patient | 76.0 | 45.6 | 50.7 | 72.1 |
| Recovery Ratio | Healthy control | 19.4 | 79.3 | 25.0 | 73.4 |
|  | noME/CFS patient | 2.7 | 98.8 | 50.0 | 69.0 |
|  | ME/CFS patient | 78.0 | 22.1 | 42.4 | 57.7 |
| **Young Adults (18-25y)**  (N = 81) | | | | | |
| Fmax Session 1 | Healthy control | 90.0 | 88.9 | 81.8 | 94.1 |
|  | noME/CFS patient | 14.3 | 90.5 | 33.3 | 76.0 |
|  | ME/CFS patient | 72.7 | 64.7 | 57.1 | 78.6 |
| Fmean Session 1 | Healthy control | 90.0 | 77.8 | 69.2 | 93.3 |
|  | noME/CFS patient | 00.0 | 100.0 | - | 75.0 |
|  | ME/CFS patient | 81.8 | 64.7 | 60.0 | 84.6 |
| FR Session 1 | Healthy control | 60.0 | 55.6 | 42.9 | 71.4 |
|  | noME/CFS patient | 00.0 | 100.0 | - | 75.0 |
|  | ME/CFS patient | 54.5 | 52.9 | 42.9 | 64.3 |
| Fmax Session 2 | Healthy control | 90.0 | 88.2 | 81.8 | 93.8 |
|  | noME/CFS patient | 28.6 | 85.0 | 40.0 | 77.3 |
|  | ME/CFS patient | 60.0 | 70.6 | 54.5 | 75.0 |
| Fmean Session 2 | Healthy control | 90.0 | 82.4 | 75.0 | 93.3 |
|  | noME/CFS patient | 28.6 | 90.0 | 50.0 | 78.3 |
|  | ME/CFS patient | 60.0 | 70.6 | 54.5 | 75.0 |
| FR Session 2 | Healthy control | 70.0 | 47.1 | 43.8 | 72.7 |
|  | noME/CFS patient | 00.0 | 100.0 | - | 74.1 |
|  | ME/CFS patient | 40.0 | 58.8 | 36.4 | 62.5 |
| Recovery Ratio | Healthy control | 60.0 | 52.9 | 42.9 | 69.2 |
|  | noME/CFS patient | 0.0 | 100.0 | - | 74.1 |
|  | ME/CFS patient | 50.0 | 52.9 | 38.5 | 64.3 |

**Supplementary Table S7.** *Classification metrics of proportional odds models assessing the diagnostic value of the seven handgrip strength indices in distinguishing noME/CFS patients from confirmed CCC-ME/CFS patients.*

| **Parameter** | **Sensitivity (%)** | **Specificity (%)** | **PPV (%)** | **NPV (%)** |
| --- | --- | --- | --- | --- |
| **All Patients**  (N = 103) | | | | |
| Fmax Session 1 | 42.5 | 80.6 | 58.6 | 68.5 |
| Fmean Session 1 | 42.5 | 79.0 | 56.7 | 68.1 |
| FR Session 1 | 42.5 | 80.3 | 58.6 | 68.1 |
| Fmax Session 2 | 46.2 | 85.2 | 66.7 | 71.2 |
| Fmean Session 2 | 43.6 | 82.0 | 60.7 | 69.4 |
| FR Session 2 | 41.0 | 80.0 | 57.1 | 67.6 |
| Recovery Ratio | 48.7 | 85.0 | 67.9 | 71.8 |
| **Female**  (N = 65) | | | | |
| Fmax Session 1 | 50.0 | 70.6 | 60.0 | 61.5 |
| Fmean Session 1 | 53.3 | 70.6 | 61.5 | 63.2 |
| FR Session 1 | 56.7 | 69.7 | 63.0 | 63.9 |
| Fmax Session 2 | 58.6 | 79.4 | 70.8 | 69.2 |
| Fmean Session 2 | 55.2 | 76.5 | 66.7 | 66.7 |
| FR Session 2 | 58.6 | 66.7 | 60.7 | 64.7 |
| Recovery Ratio | 62.1 | 72.7 | 66.7 | 68.6 |
| **Male**  (N = 38) | | | | |
| Fmax Session 1 | 0.0 | 100.0 | - | 73.7 |
| Fmean Session 1 | 0.0 | 100.0 | - | 73.7 |
| FR Session 1 | 10.0 | 100.0 | 100.0 | 75.7 |
| Fmax Session 2 | 0.0 | 100.0 | - | 73.0 |
| Fmean Session 2 | 0.0 | 100.0 | - | 73.0 |
| FR Session 2 | 0.0 | 100.0 | - | 73.0 |
| Recovery Ratio | 0.0 | 100.0 | - | 73.0 |
| **Children (10-12y)**  (N = 9) | | | | |
| Fmax Session 1 | 100.0 | 100.0 | 100.0 | 100.0 |
| Fmean Session 1 | 100.0 | 100.0 | 100.0 | 100.0 |
| FR Session 1 | 50.0 | 100.0 | 100.0 | 87.5 |
| Fmax Session 2 | 100.0 | 100.0 | 100.0 | 100.0 |
| Fmean Session 2 | 100.0 | 100.0 | 100.0 | 100.0 |
| FR Session 2 | 100.0 | 100.0 | 100.0 | 100.0 |
| Recovery Ratio | 100.0 | 100.0 | 100.0 | 100.0 |
| **Adolescents (13-17y)**  (N = 63) | | | | |
| Fmax Session 1 | 29.2 | 87.2 | 58.3 | 66.7 |
| Fmean Session 1 | 29.2 | 87.2 | 58.3 | 66.7 |
| FR Session 1 | 37.5 | 81.6 | 56.2 | 67.4 |
| Fmax Session 2 | 29.2 | 86.8 | 58.3 | 66.0 |
| Fmean Session 2 | 33.3 | 84.2 | 57.1 | 66.7 |
| FR Session 2 | 33.3 | 83.8 | 57.1 | 66.0 |
| Recovery Ratio | 29.2 | 89.2 | 63.6 | 66.0 |
| **Young Adults (18-25y)**  (N = 31) | | | | |
| Fmax Session 1 | 78.6 | 75.0 | 73.3 | 80.0 |
| Fmean Session 1 | 78.6 | 81.2 | 78.6 | 81.2 |
| FR Session 1 | 78.6 | 87.5 | 84.6 | 82.4 |
| Fmax Session 2 | 84.6 | 93.8 | 91.7 | 88.2 |
| Fmean Session 2 | 76.9 | 87.5 | 83.3 | 82.4 |
| FR Session 2 | 76.9 | 81.2 | 76.9 | 81.2 |
| Recovery Ratio | 69.2 | 81.2 | 75.0 | 76.5 |
| Abbreviations: **PPV** – positive predictive value, **NPV** – negative predictive value. | | | | |
