# Supplementary material for: Handgrip strength in children, adolescents, and young adults with suspected myalgic encephalomyelitis/chronic fatigue syndrome
Source: J Transl Med. 2026 Jul 15;24:947. doi: 10.1186/s12967-026-08654-5 (PMC13393269; doi:10.1186/s12967-026-08654-5)
Supplement: Supplementary file 2 — Supplementary Material 2: Supplementary Figure 1 [file 12967_2026_8654_MOESM2_ESM.docx]

**
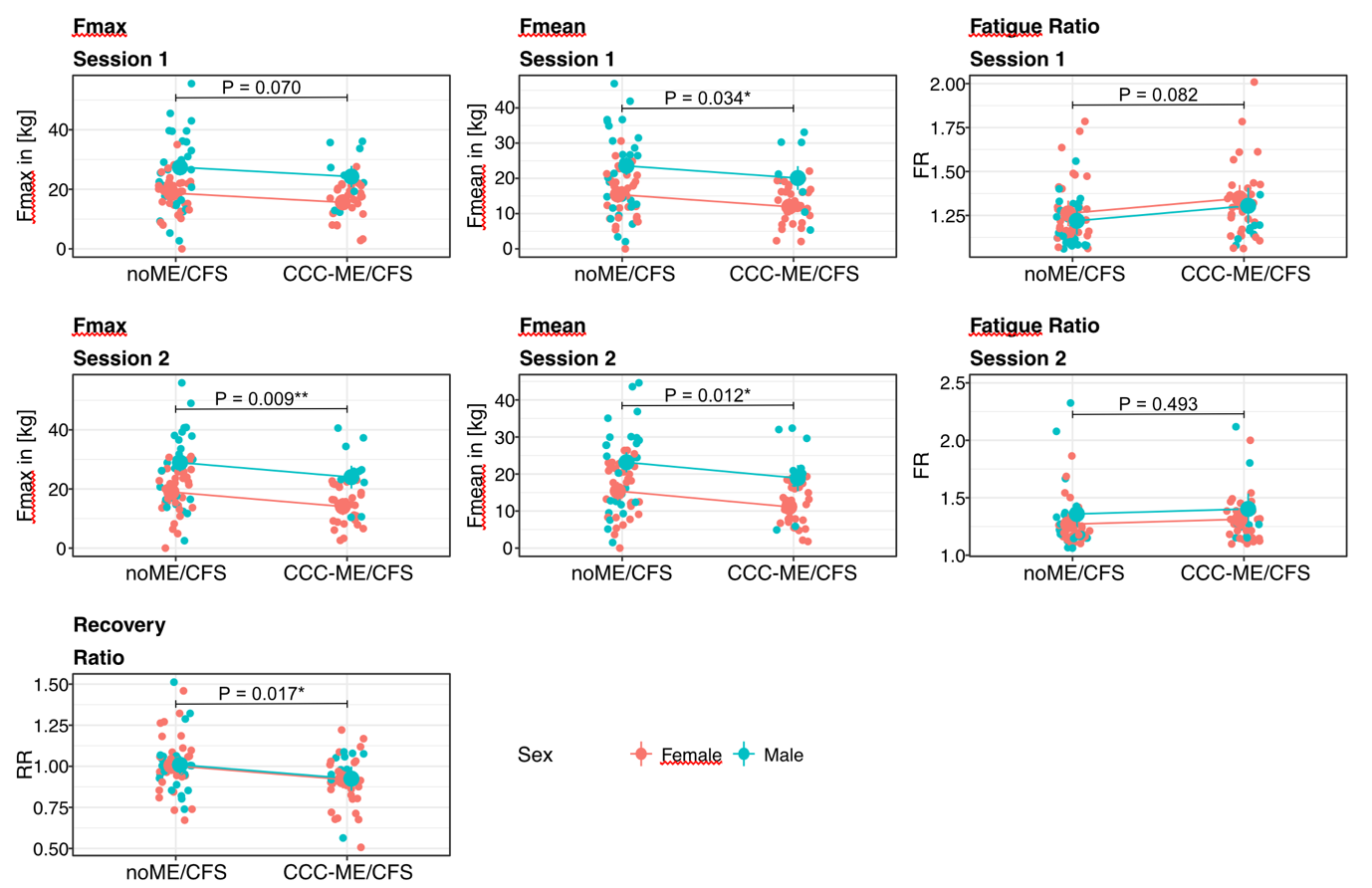
**

**Supplementary Figure S1.** *Marginal means of all handgrip strength (HGS) indices comparing noME/CFS patients with confirmed CCC-ME/CFS patients, adjusted for age and body mass index (BMI), for male (green) and female (red) participants. Estimates were obtained from linear models that included group, sex, age, and BMI as independent variables, and each HGS index as the dependent variable. Horizontal lines indicate significant differences between groups* *and are indicated as follows: P < 0.1 (.), P < 0.05 (*), P < 0.01 (**), P < 0.001 (***). Outliers above the fatigue ratio (FR) cutoffs were excluded from the plot for clarity, but were retained in the statistical analyses. These included, for FR in Session 1 (cutoff = 2.0): 1 in noME/CFS patients and 2 in CCC-ME/CFS patients; and for FR in Session 2 (cutoff = 2.5): 1 in noME/CFS patients and 0 in CCC-ME/CFS patients.*
